# Supplementary figures and images for: In silico design and validation of high-affinity RNA aptamers for SARS-CoV-2 comparable to neutralizing antibodies
Source: eLife. 2026 Jul 14;14:RP107785. doi: 10.7554/eLife.107785 (PMC13368180; doi:10.7554/eLife.107785)

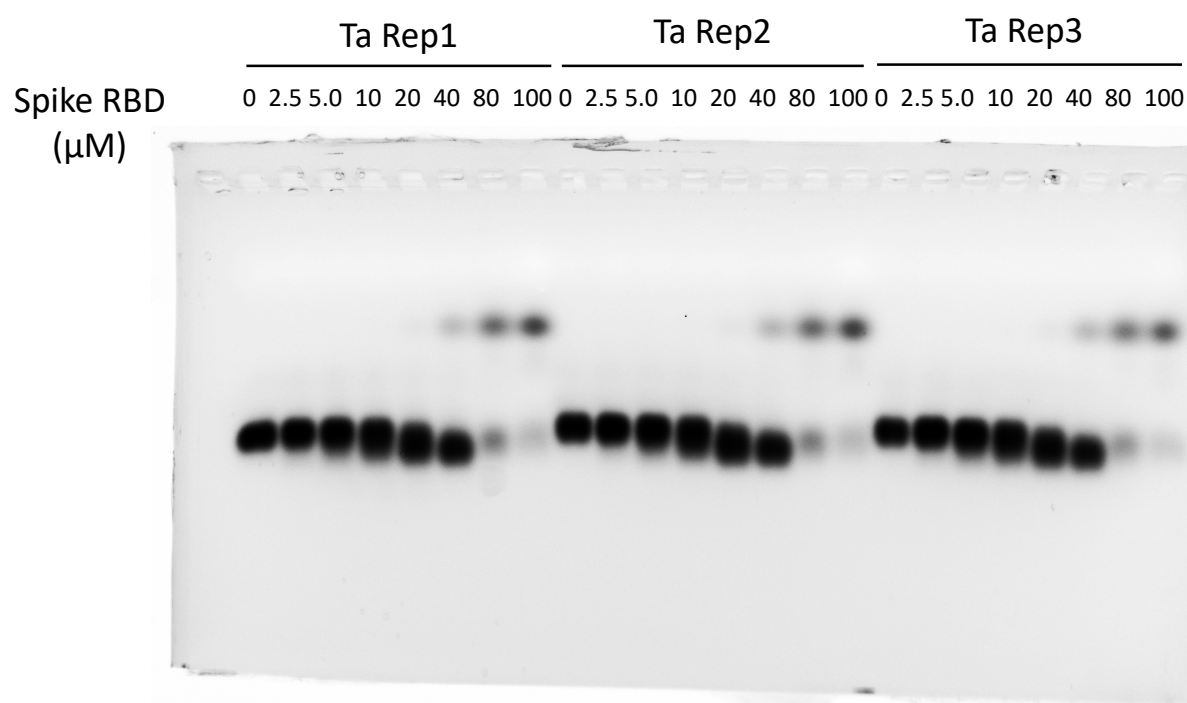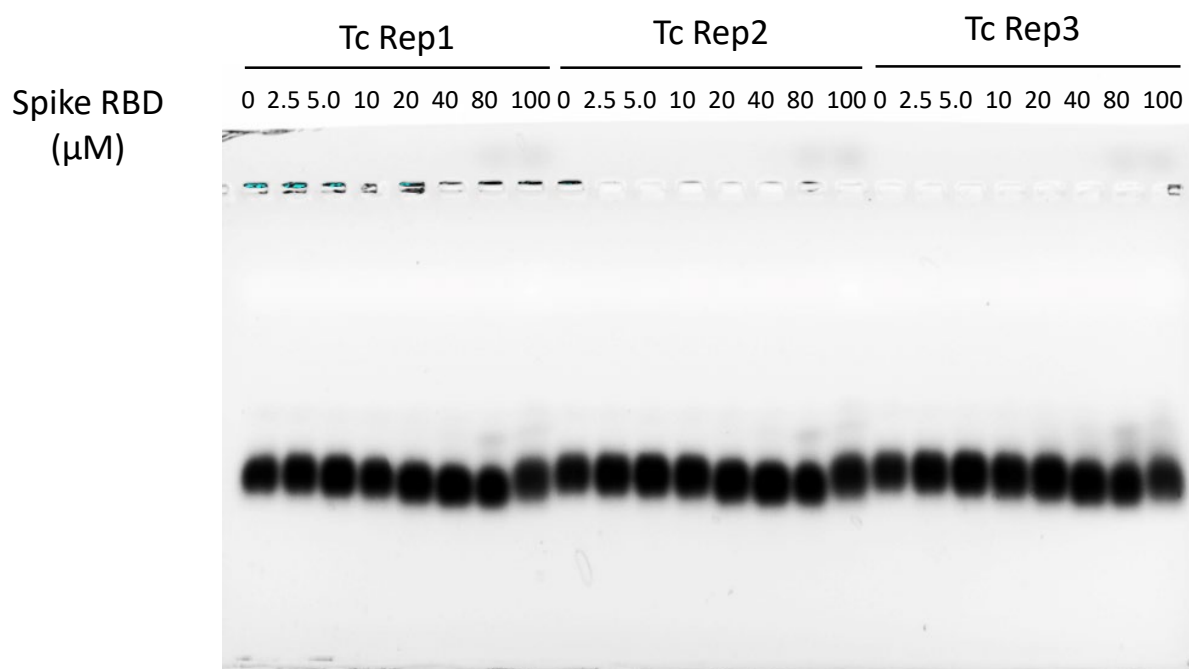

Supplement: Figure 2—source data 1. [file elife-107785-fig2-data1.pdf]

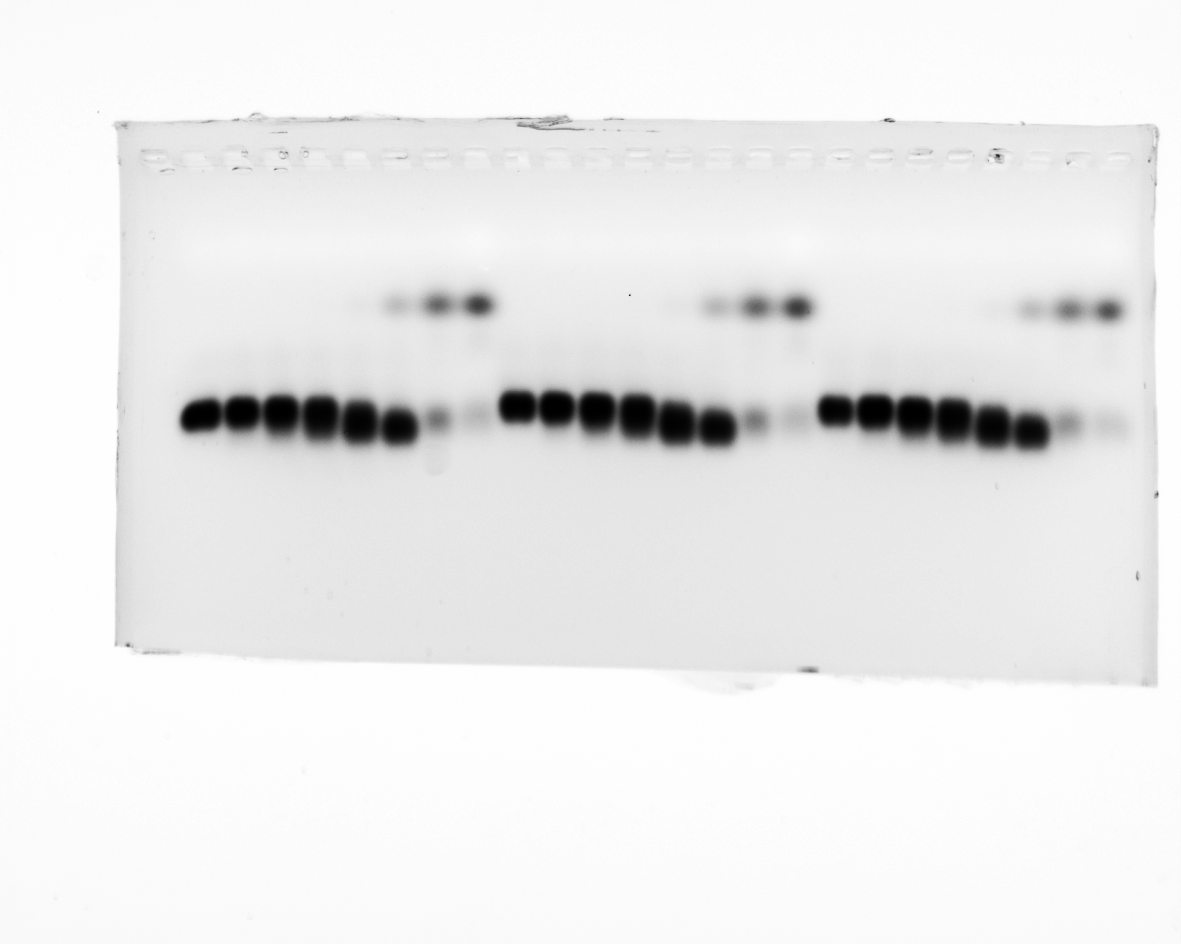

Supplement: Figure 2—source data 2. [file elife-107785-fig2-data2.zip › Fig2F-Ta.tif]

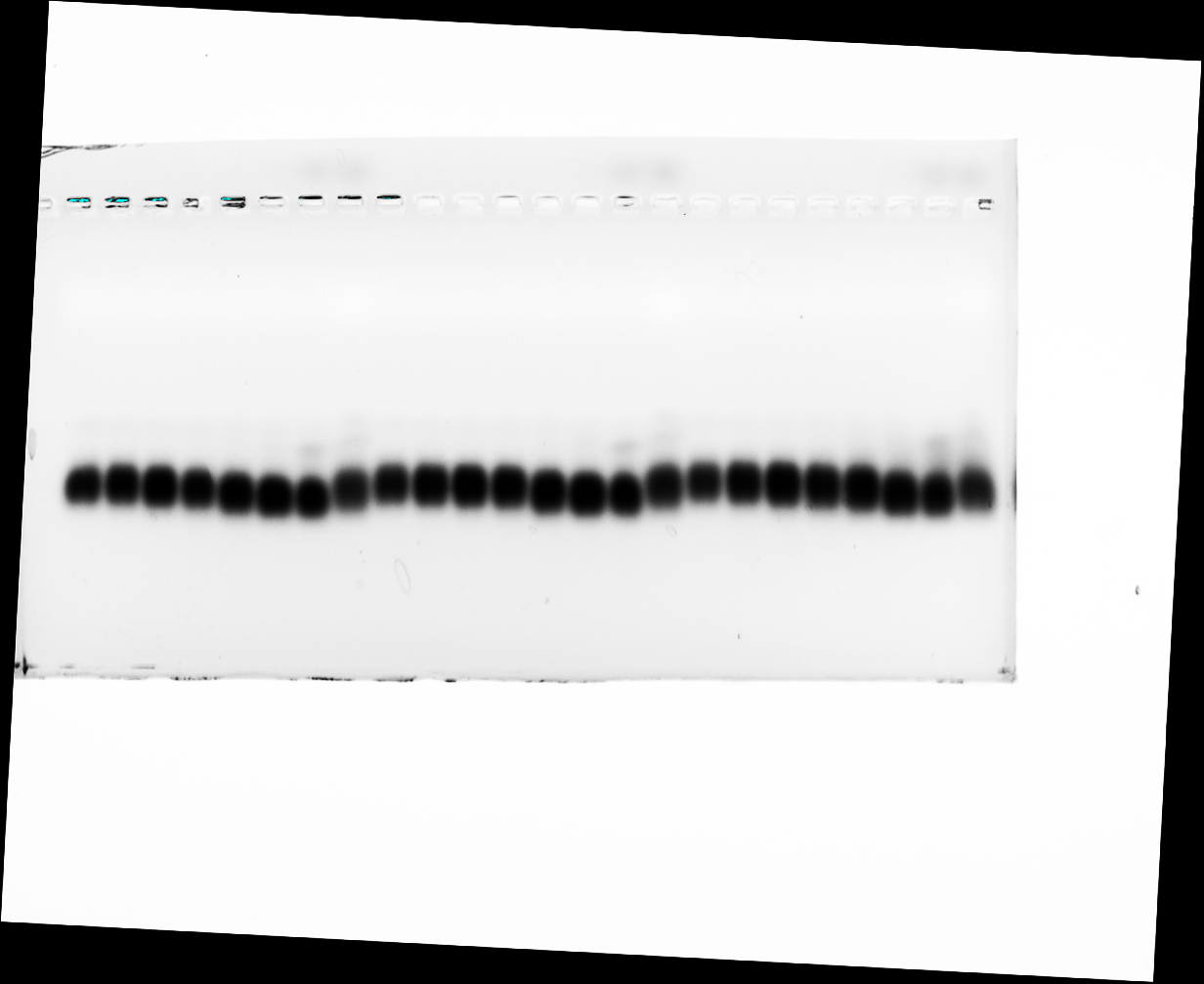

Supplement: Figure 2—source data 2. [file elife-107785-fig2-data2.zip › Fig2F-Tc.tif]

|                          |   |   |   |
|--------------------------|---|---|---|
| RBD                      | + | - | + |
| Antibody<br>(40592-R001) | - | + | + |

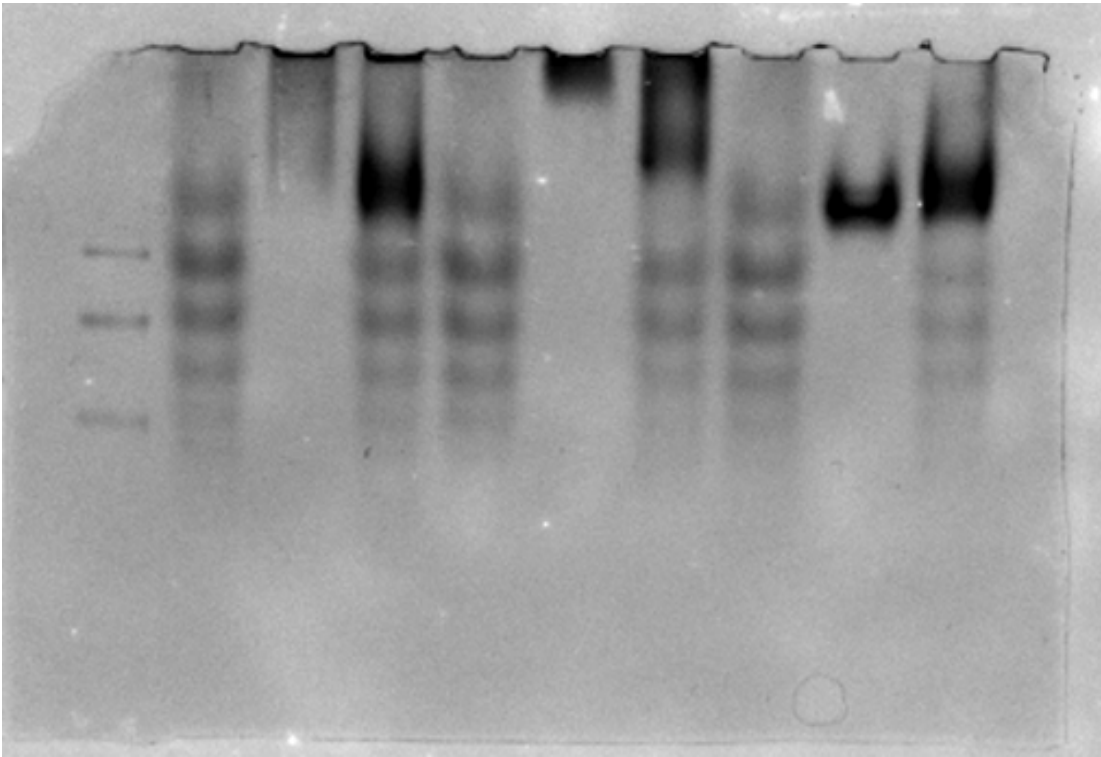

|                          |   |   |   |   |
|--------------------------|---|---|---|---|
| Ta                       | + | + | + | + |
| RBD                      | - | + | - | + |
| Antibody<br>(40592-R001) | - | - | + | + |

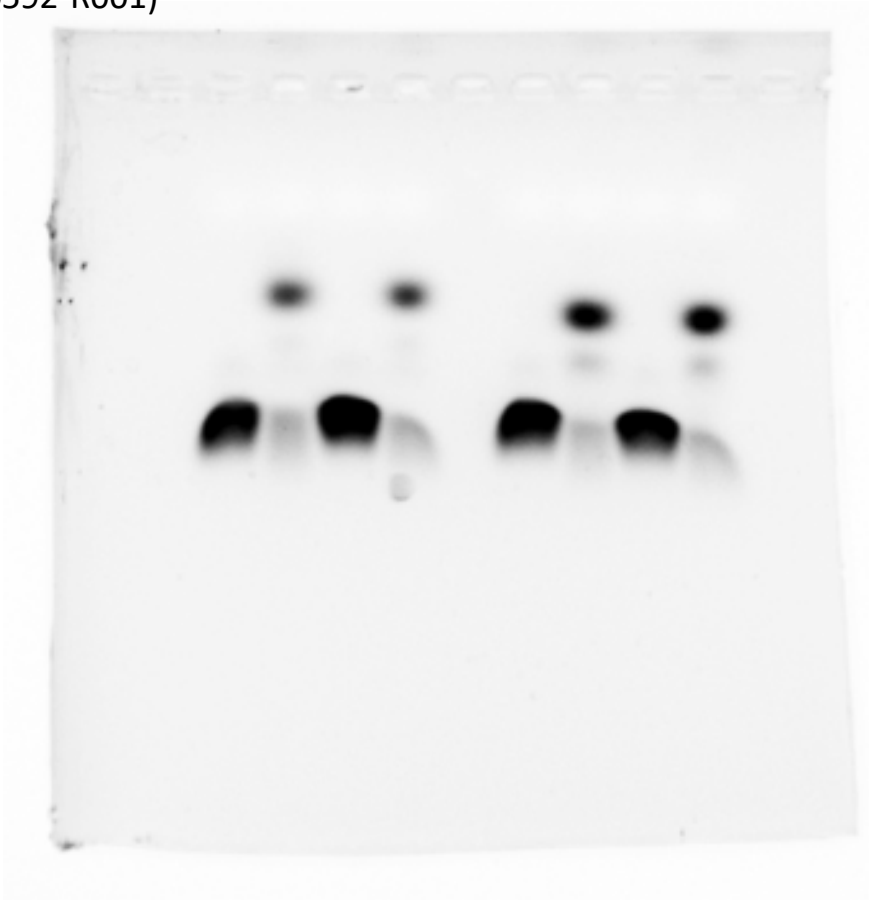

Supplement: Figure 3—source data 1. [file elife-107785-fig3-data1.pdf]

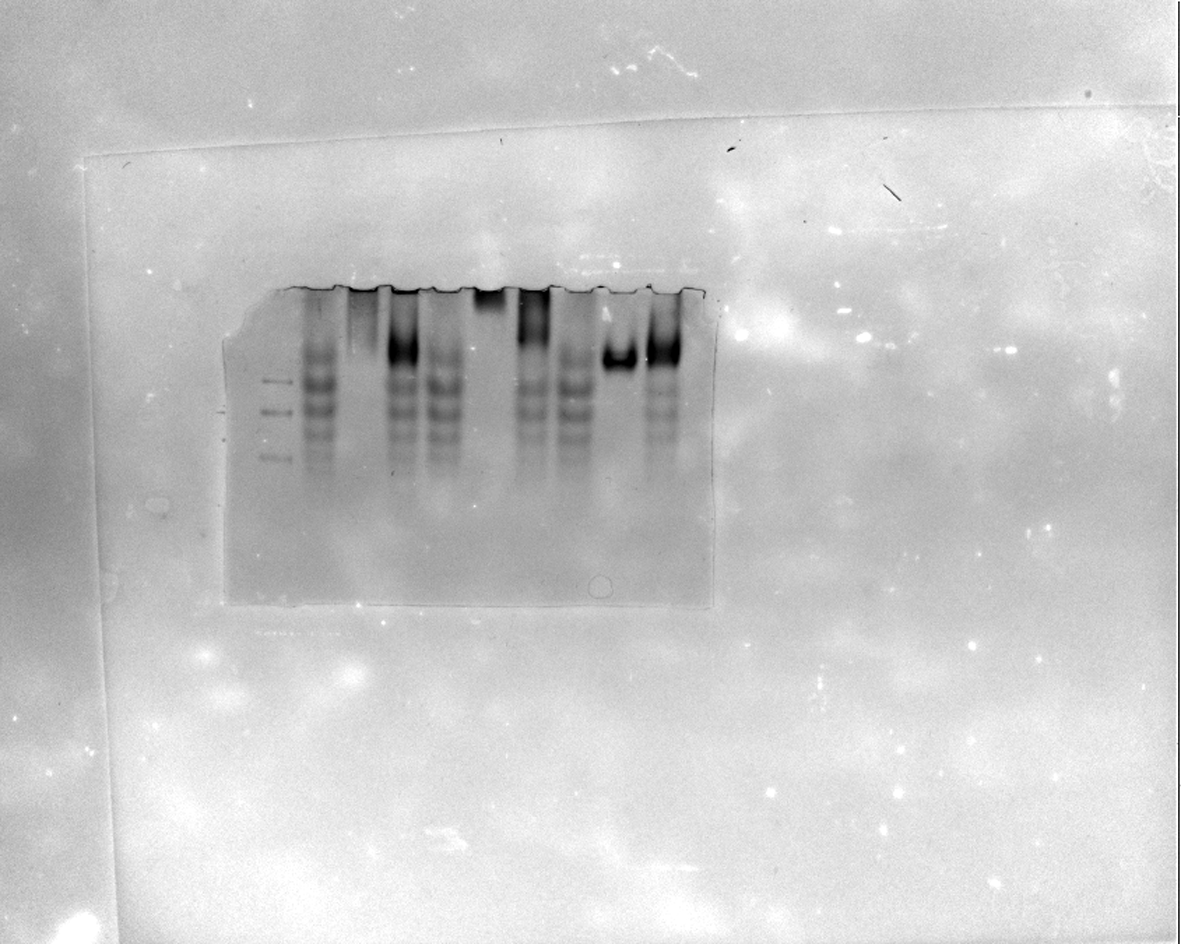

Supplement: Figure 3—source data 2. [file elife-107785-fig3-data2.zip › Fig3D.tif]

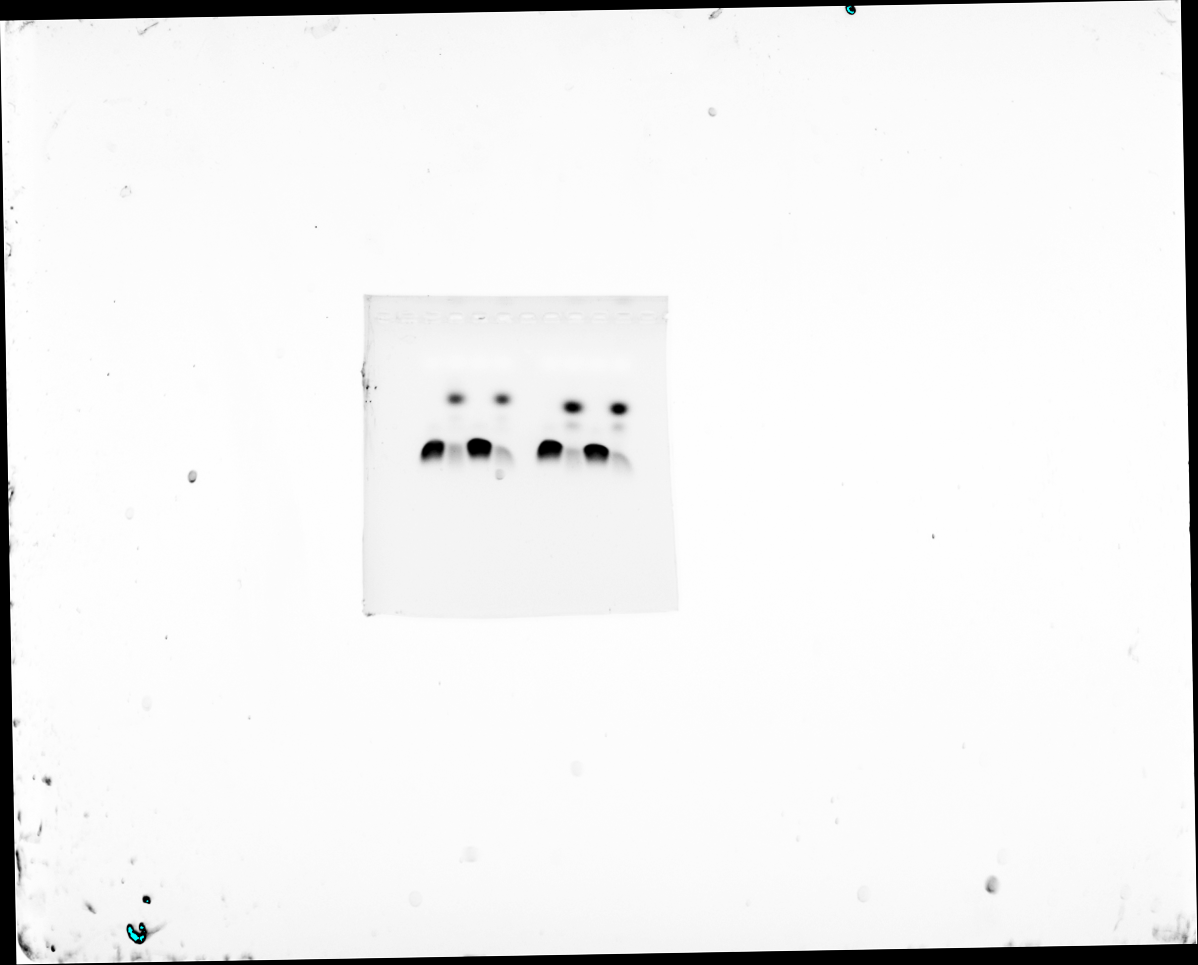

Supplement: Figure 3—source data 2. [file elife-107785-fig3-data2.zip › Fig3E.tif]

|     |           |   |           |   |             |   |             |   |             |   |             |   |             |   |
|-----|-----------|---|-----------|---|-------------|---|-------------|---|-------------|---|-------------|---|-------------|---|
|     | <u>Ta</u> |   | <u>Tc</u> |   | <u>G34C</u> |   | <u>G34U</u> |   | <u>G34A</u> |   | <u>C23G</u> |   | <u>C23A</u> |   |
| RBD | -         | + | -         | + | -           | + | -           | + | -           | + | -           | + | -           | + |

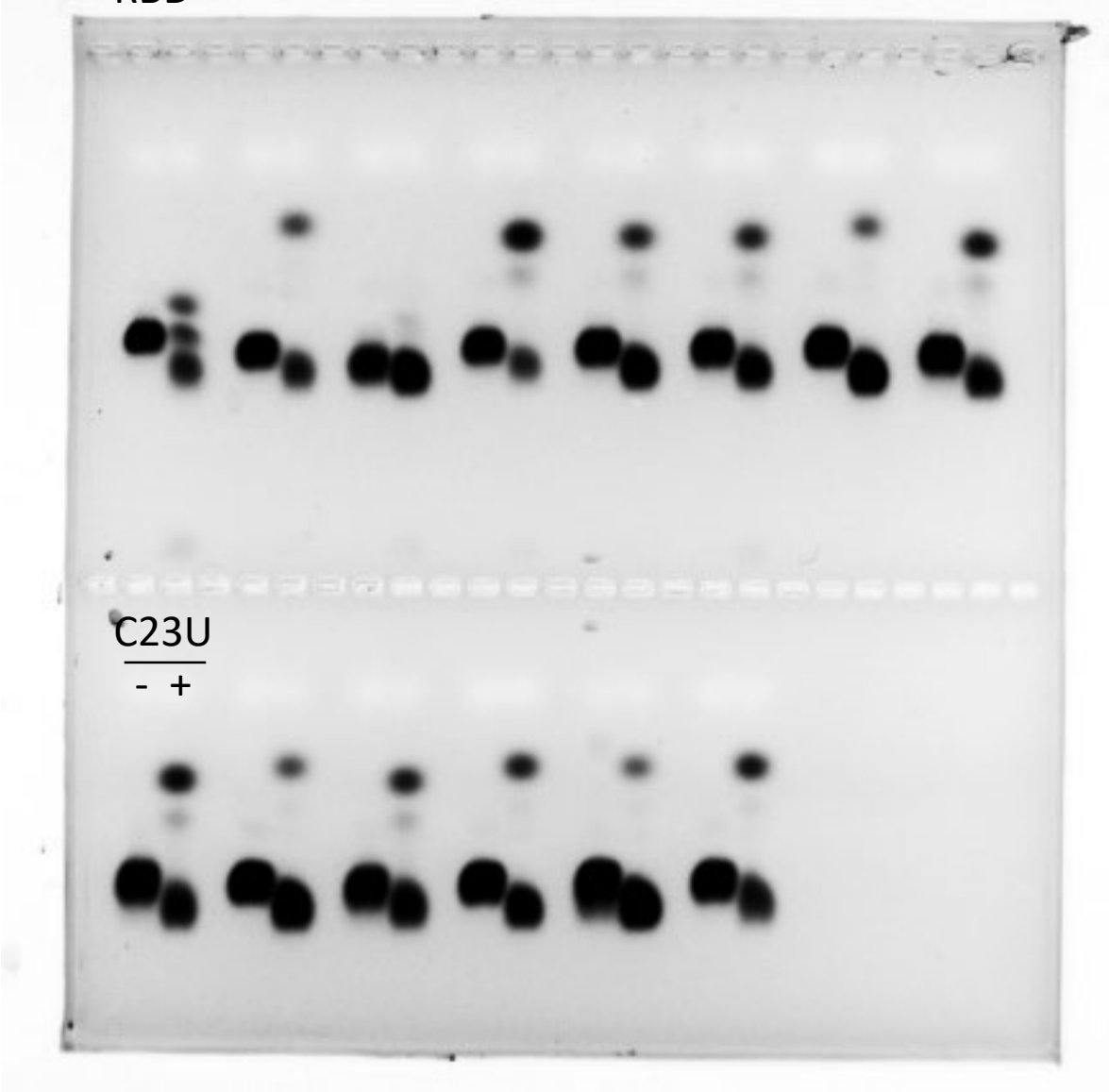

Supplement: Figure 4—source data 1. [file elife-107785-fig4-data1.pdf]

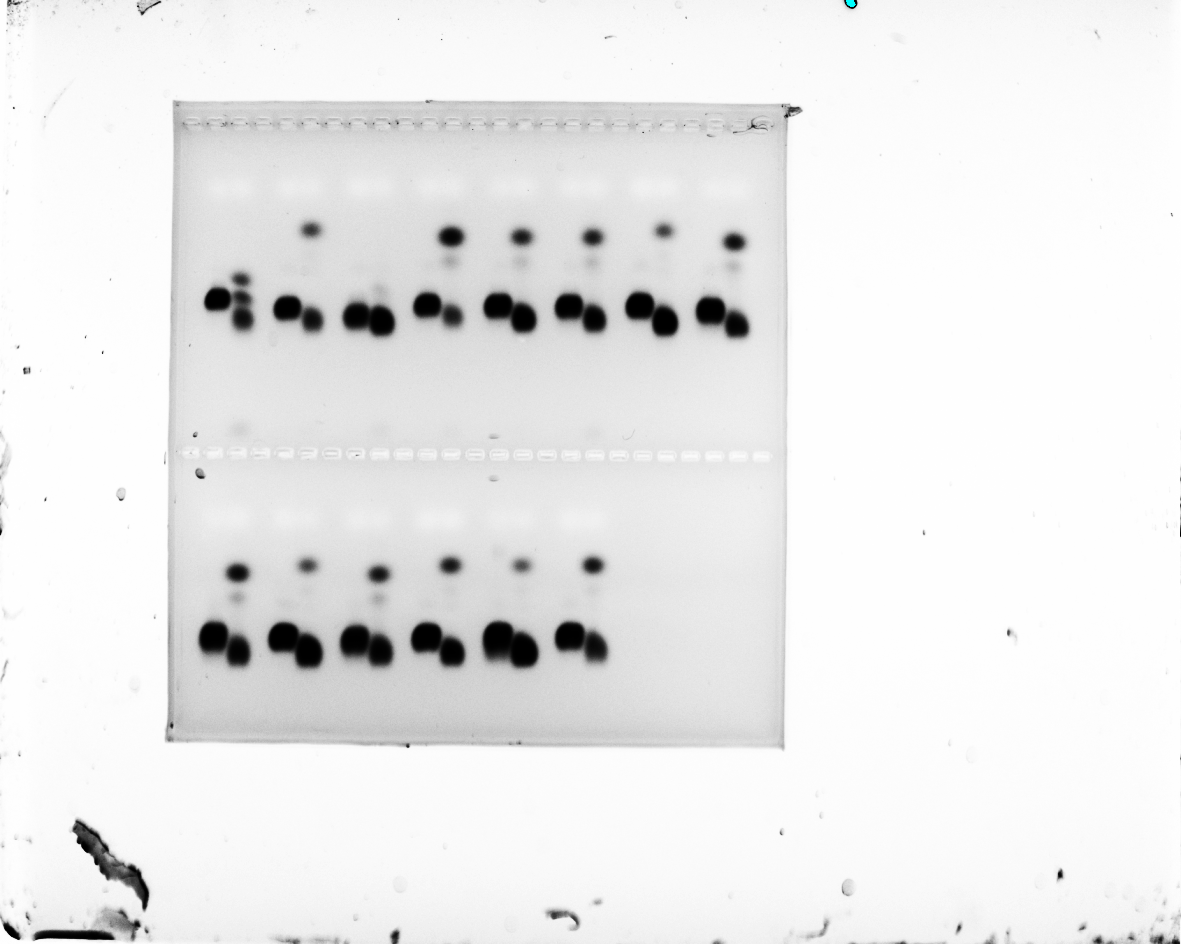

Supplement: Figure 4—source data 2. [file elife-107785-fig4-data2.zip › Fig4E.tif]

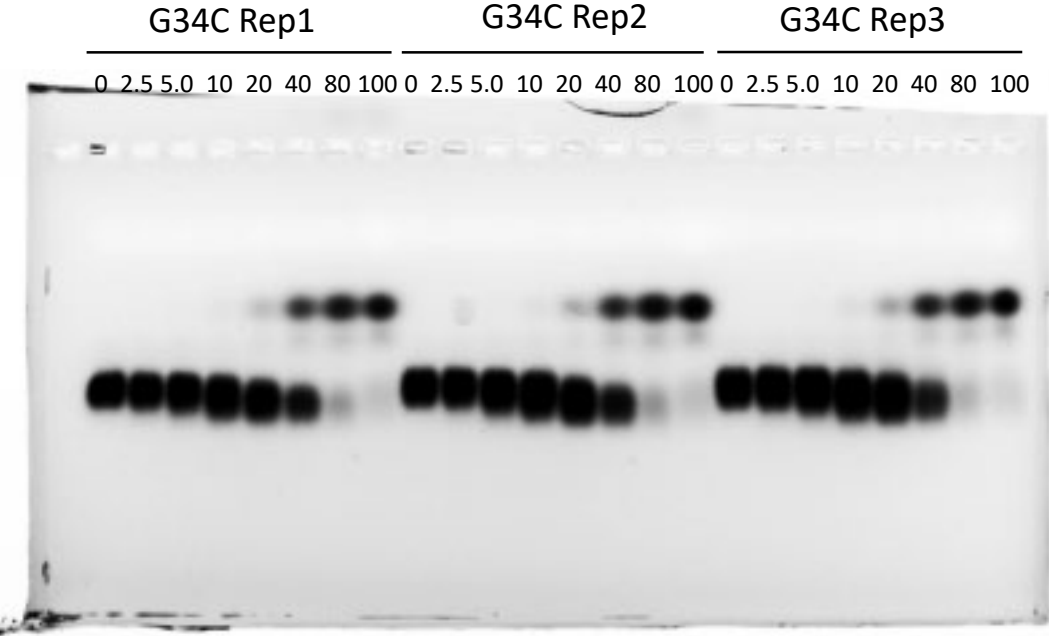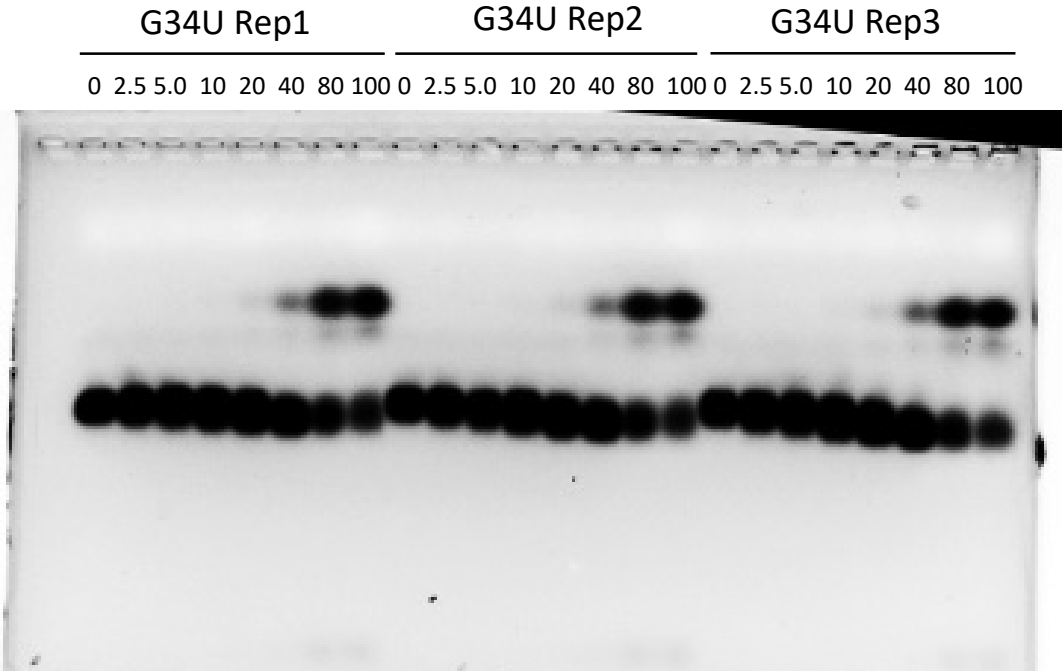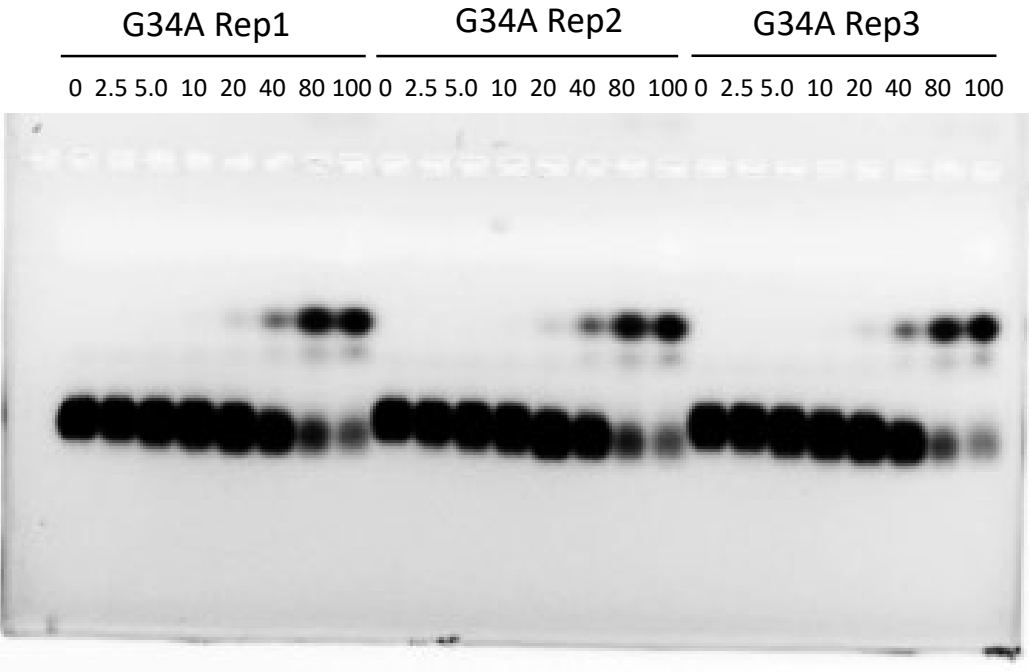

| C23G Rep1 |     |     |    |    |    |    |     |   |     | C23G Rep2 |    |    |    |    |     |   |     |     |    | C23G Rep3 |    |    |     |  |  |  |  |  |  |
|-----------|-----|-----|----|----|----|----|-----|---|-----|-----------|----|----|----|----|-----|---|-----|-----|----|-----------|----|----|-----|--|--|--|--|--|--|
| 0         | 2.5 | 5.0 | 10 | 20 | 40 | 80 | 100 | 0 | 2.5 | 5.0       | 10 | 20 | 40 | 80 | 100 | 0 | 2.5 | 5.0 | 10 | 20        | 40 | 80 | 100 |  |  |  |  |  |  |

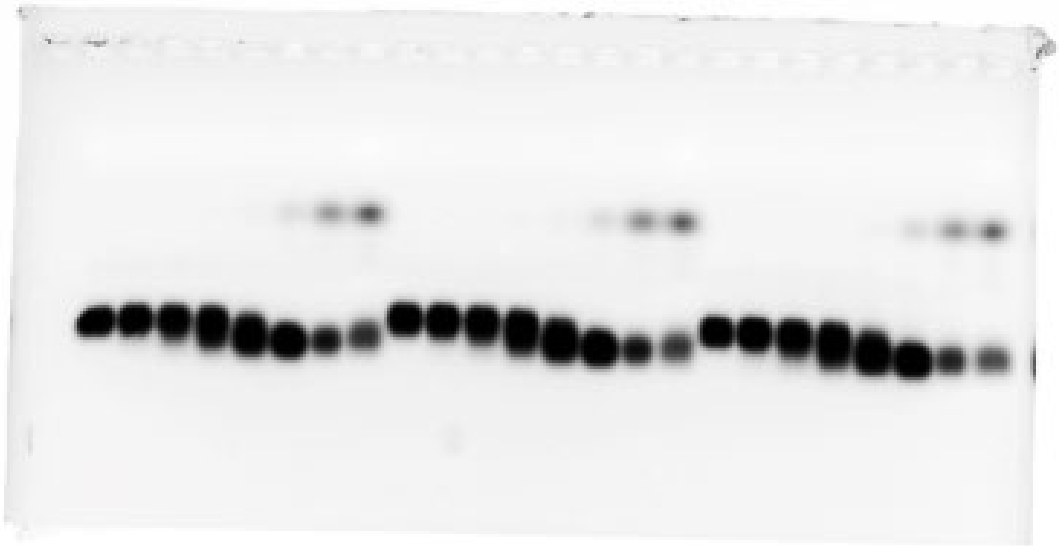

| C23A Rep1 |     |     |    |    |    |    |     | C23A Rep2 |     |     |    |    |    |    |     | C23A Rep3 |     |     |    |    |    |    |     |
|-----------|-----|-----|----|----|----|----|-----|-----------|-----|-----|----|----|----|----|-----|-----------|-----|-----|----|----|----|----|-----|
| 0         | 2.5 | 5.0 | 10 | 20 | 40 | 80 | 100 | 0         | 2.5 | 5.0 | 10 | 20 | 40 | 80 | 100 | 0         | 2.5 | 5.0 | 10 | 20 | 40 | 80 | 100 |

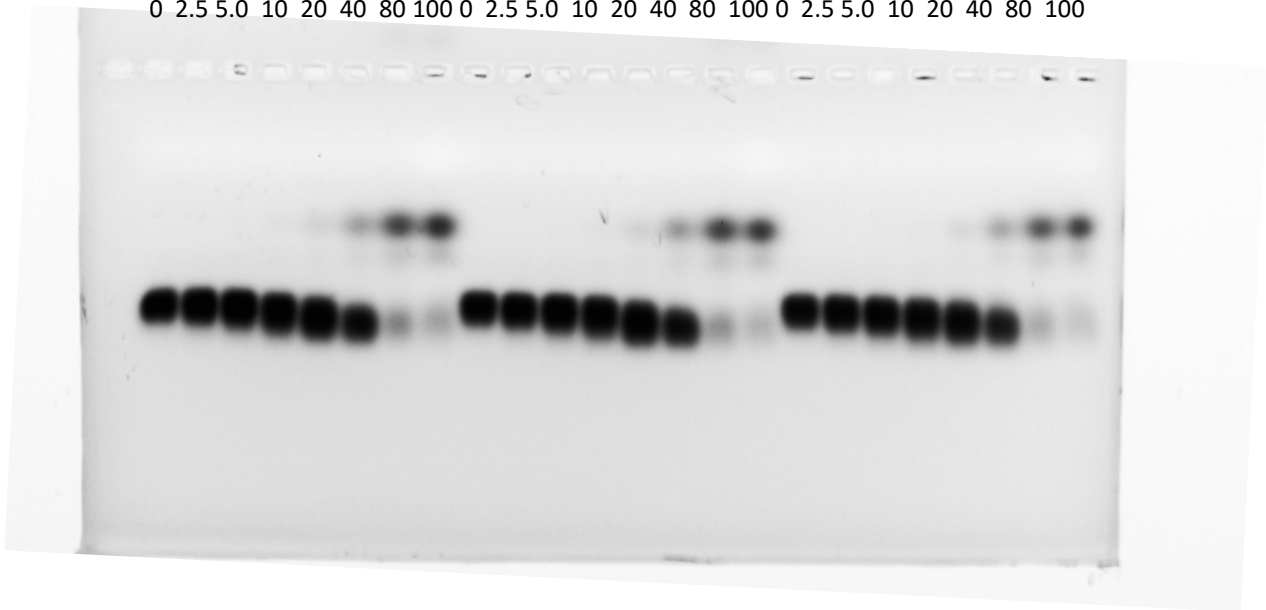

| C23U Rep1 |     |     |    |    |    |    |     | C23U Rep2 |     |     |    |    |    |    |     | C23U Rep3 |     |     |    |    |    |    |     |
|-----------|-----|-----|----|----|----|----|-----|-----------|-----|-----|----|----|----|----|-----|-----------|-----|-----|----|----|----|----|-----|
| 0         | 2.5 | 5.0 | 10 | 20 | 40 | 80 | 100 | 0         | 2.5 | 5.0 | 10 | 20 | 40 | 80 | 100 | 0         | 2.5 | 5.0 | 10 | 20 | 40 | 80 | 100 |

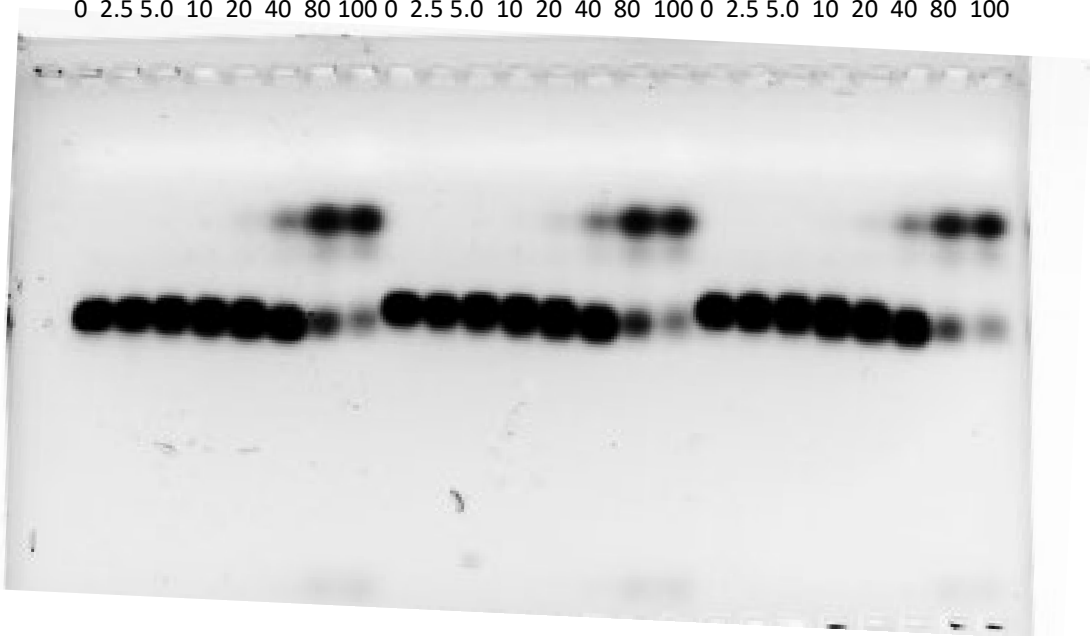

Supplement: Figure 4—figure supplement 2—source data 1. [file elife-107785-fig4-figsupp2-data1.pdf]

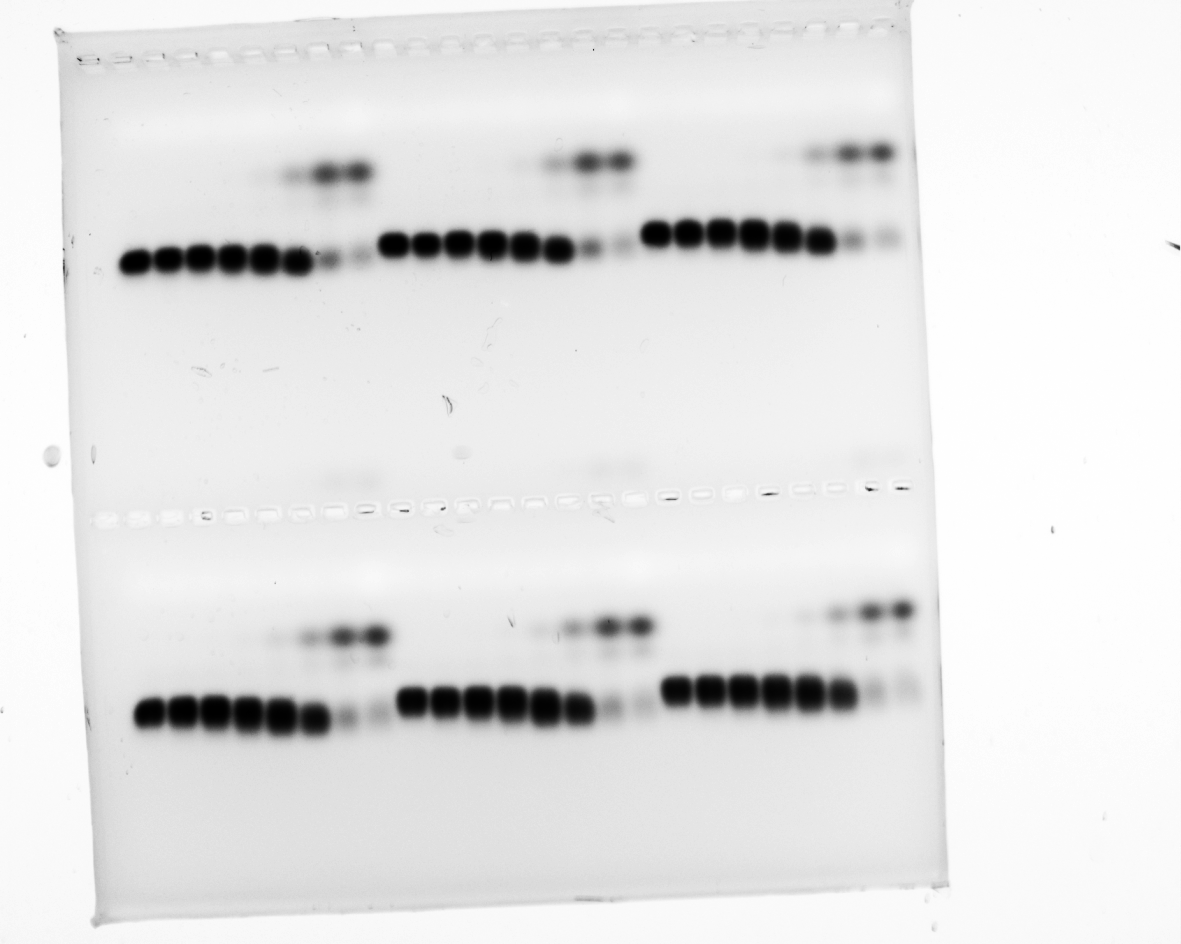

Supplement: Figure 4—figure supplement 2—source data 2. [file elife-107785-fig4-figsupp2-data2.zip › FigS9-C23A-bottom lanes.tif]

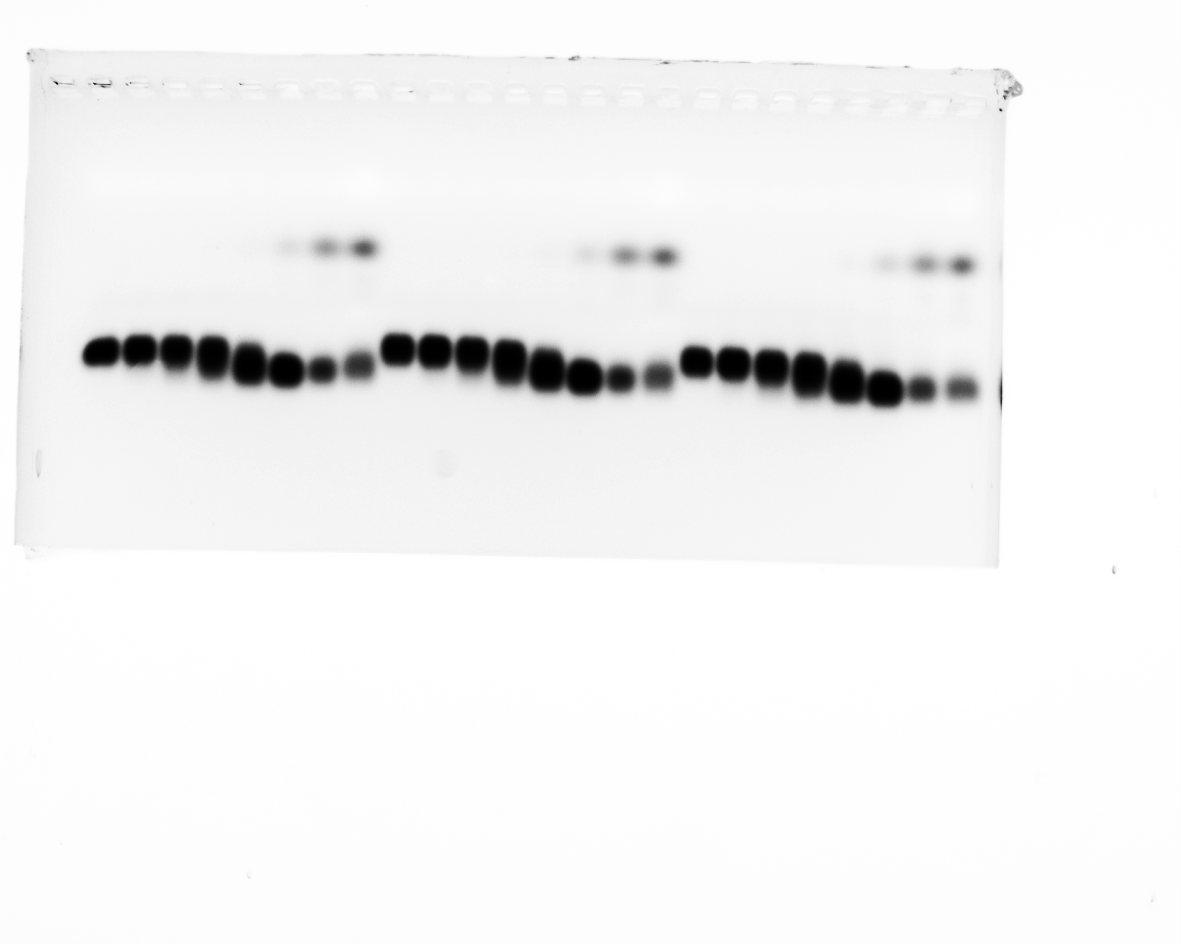

Supplement: Figure 4—figure supplement 2—source data 2. [file elife-107785-fig4-figsupp2-data2.zip › FigS9-C23G.tif]

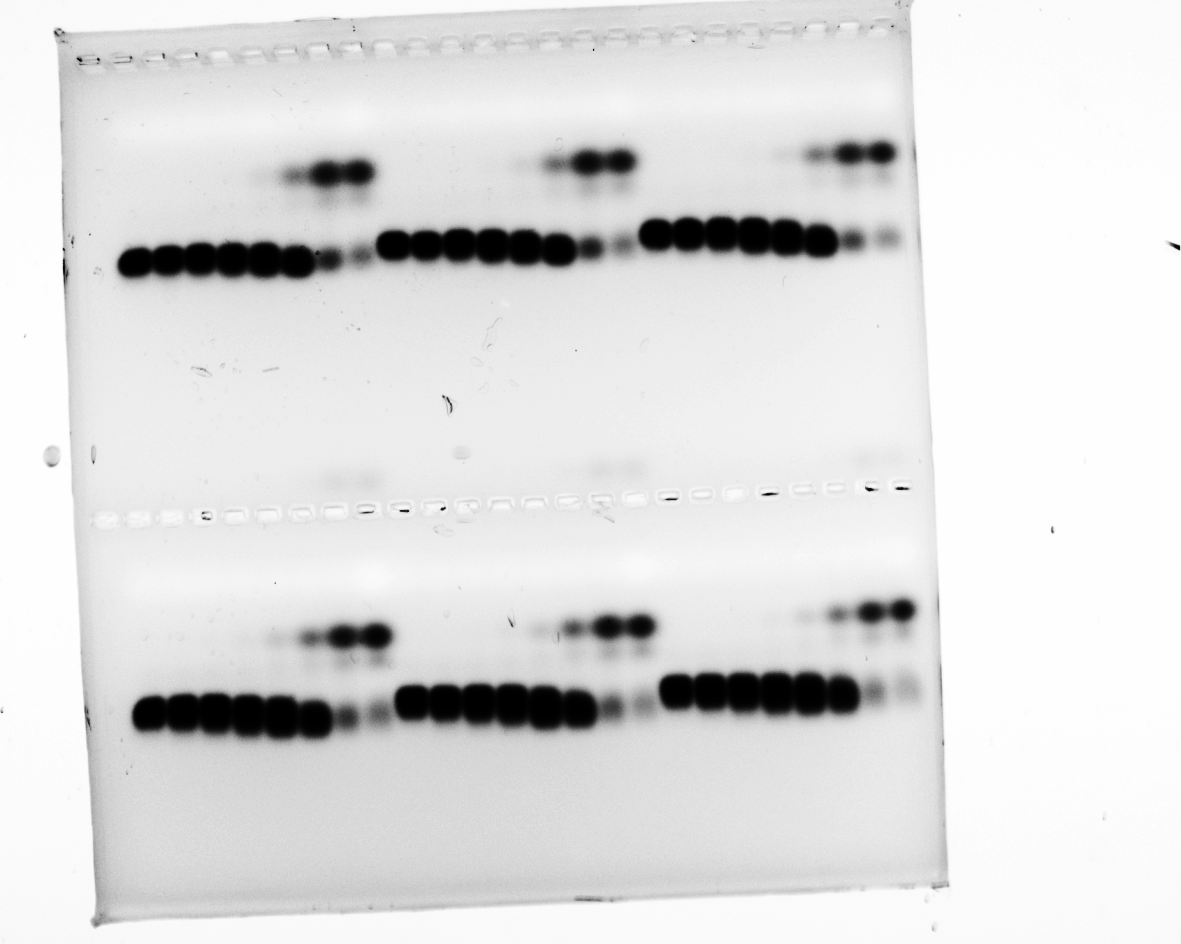

Supplement: Figure 4—figure supplement 2—source data 2. [file elife-107785-fig4-figsupp2-data2.zip › FigS9-C23U-up lanes.tif]

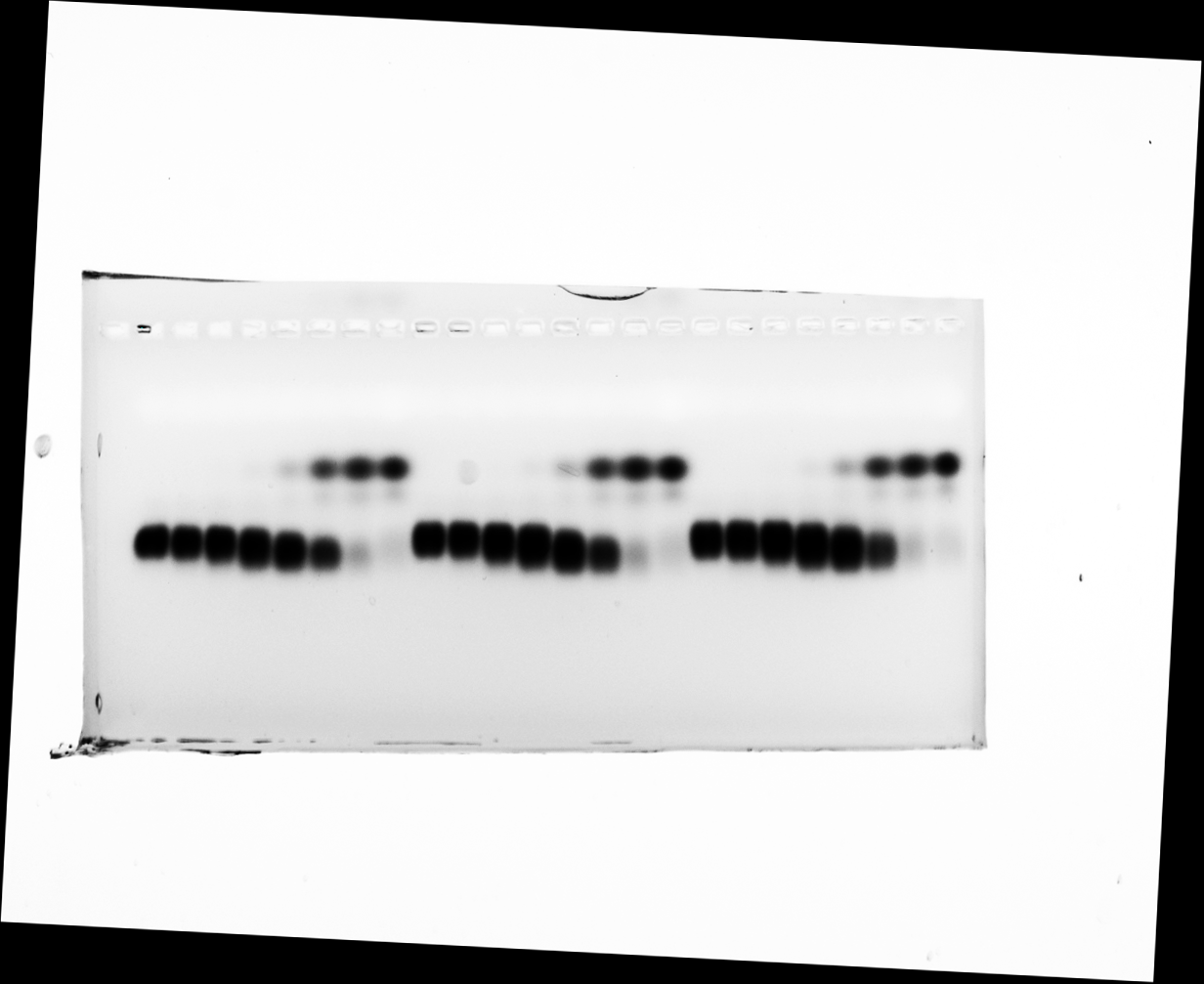

Supplement: Figure 4—figure supplement 2—source data 2. [file elife-107785-fig4-figsupp2-data2.zip › FigS9-G34C.tif]

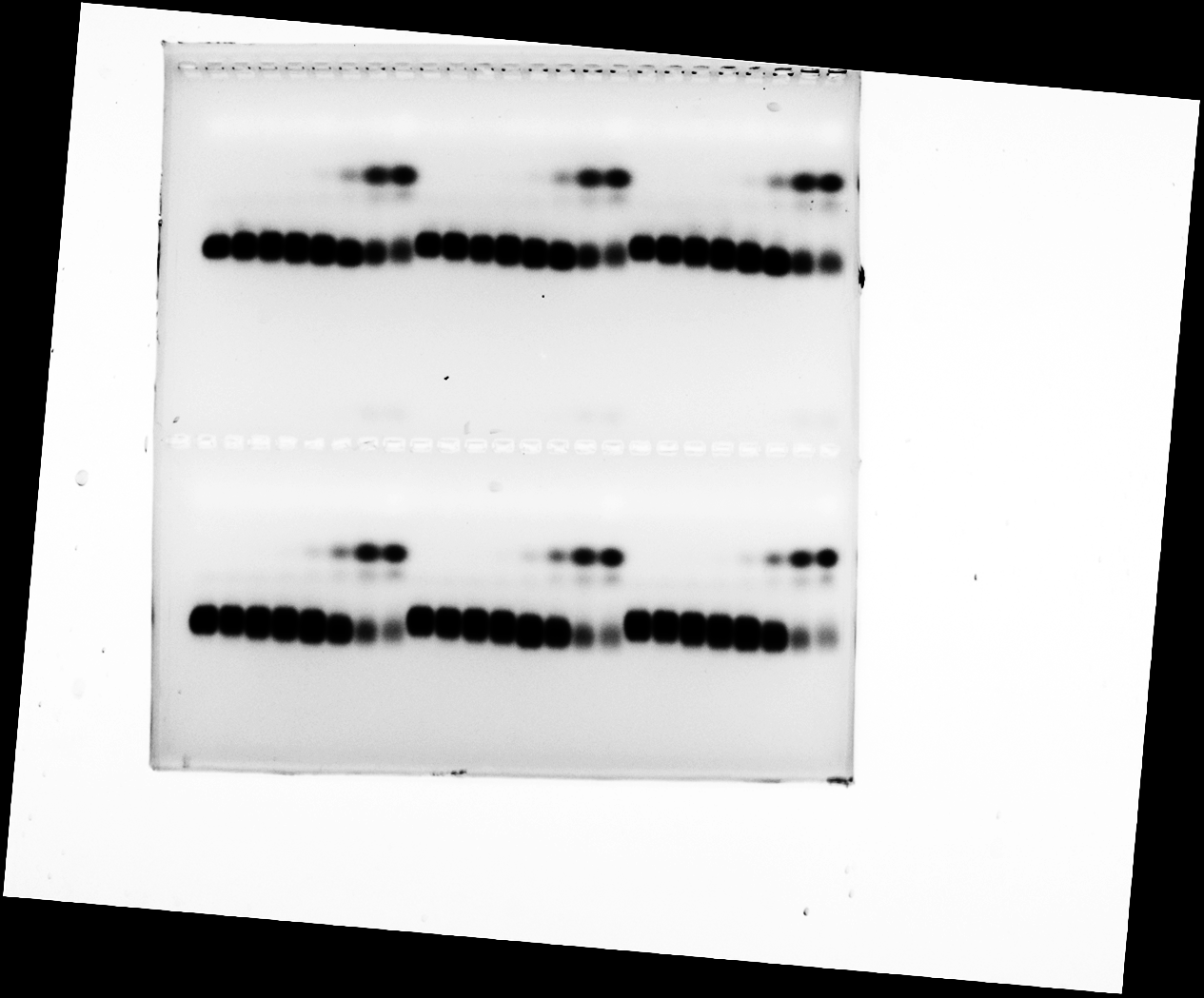

Supplement: Figure 4—figure supplement 2—source data 2. [file elife-107785-fig4-figsupp2-data2.zip › FigS9-G34U-up lanes+G34A-bottom lanes.tif]

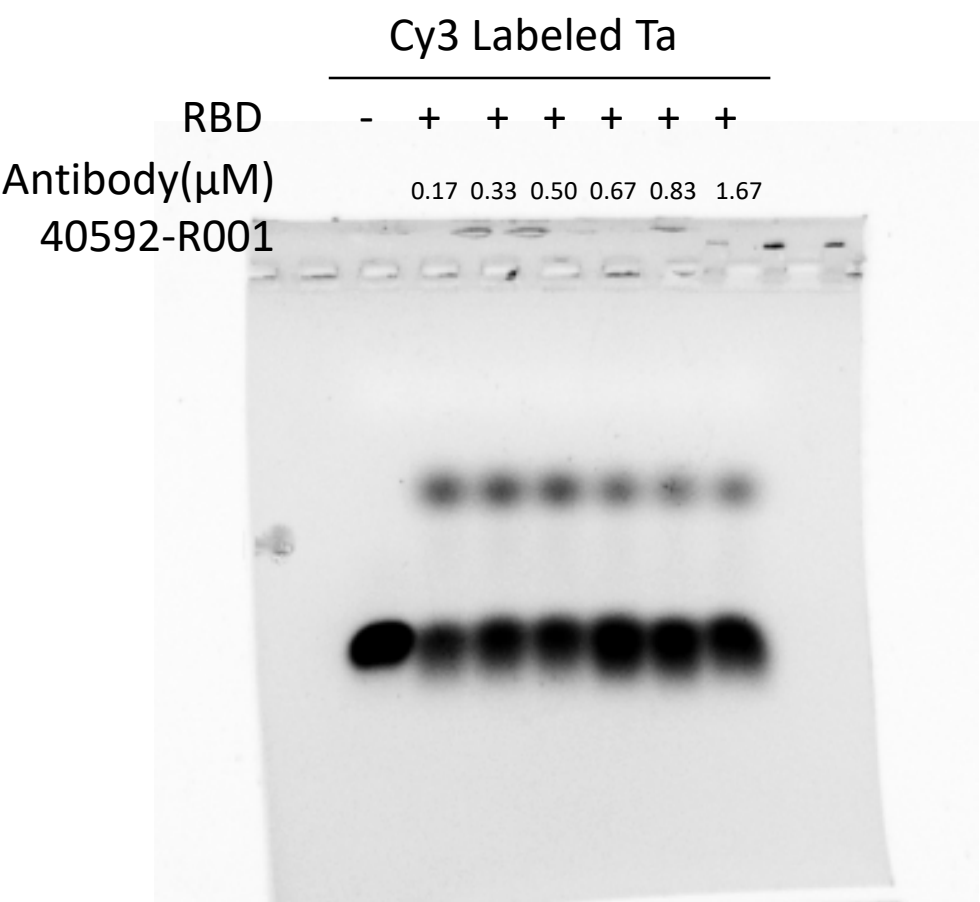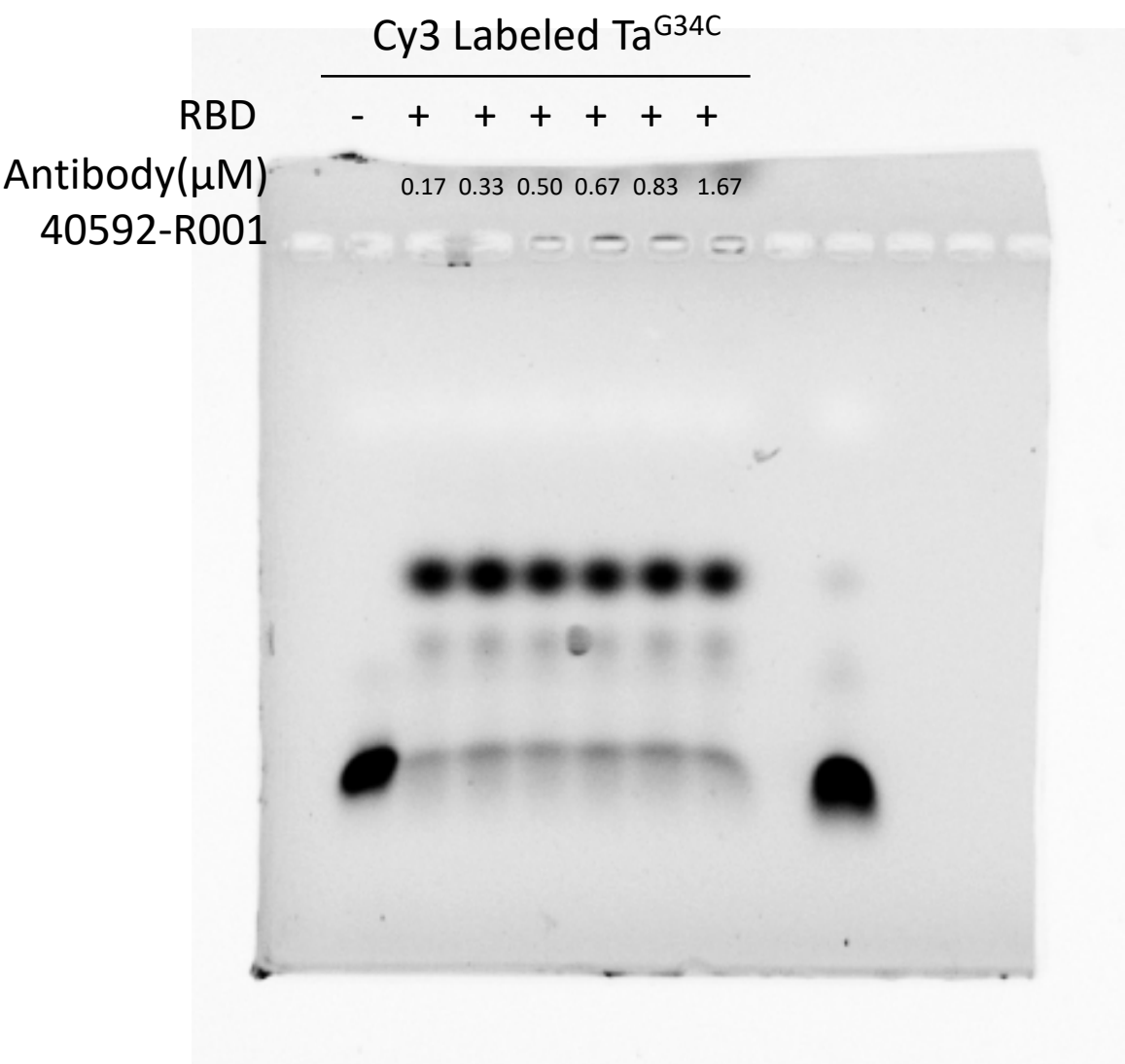

Supplement: Figure 5—source data 1. [file elife-107785-fig5-data1.pdf]

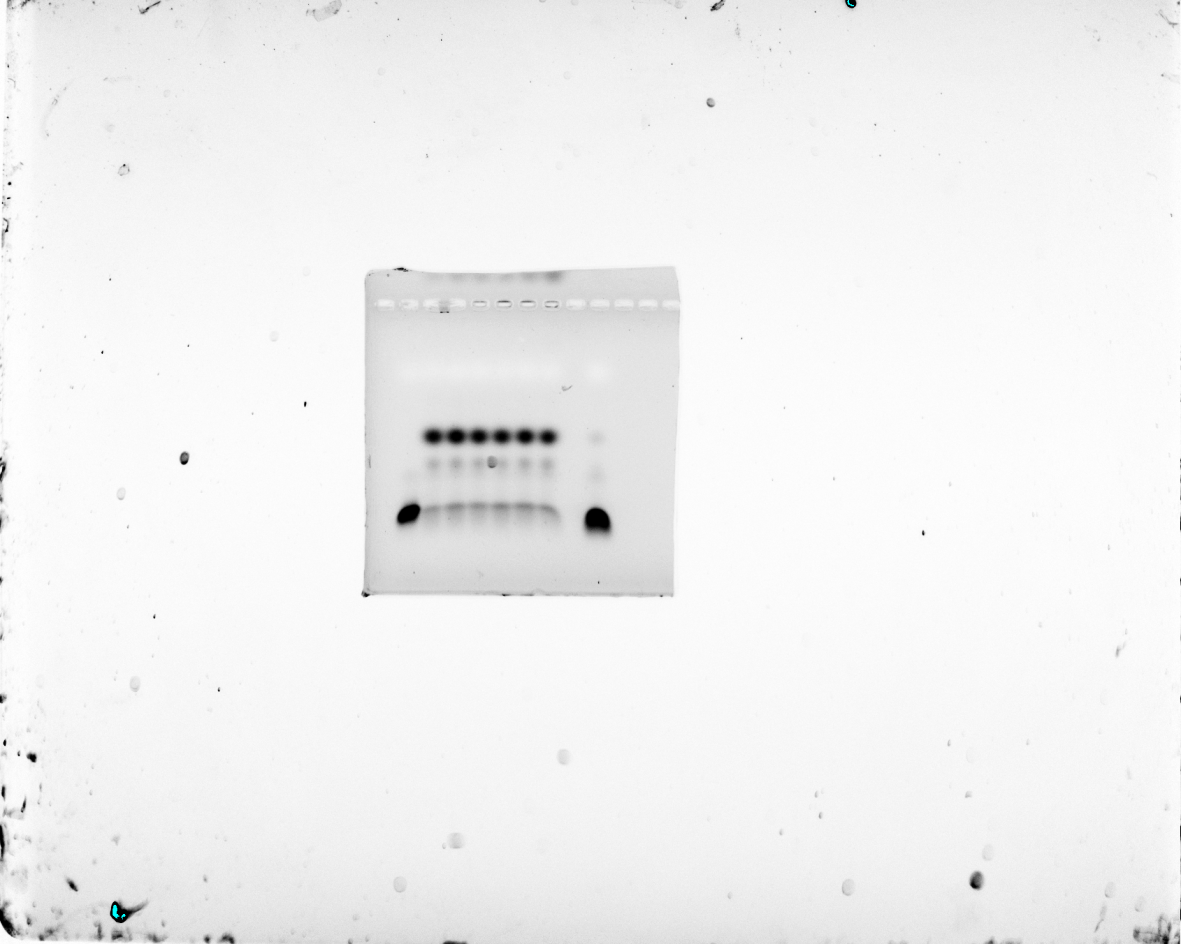

Supplement: Figure 5—source data 2. [file elife-107785-fig5-data2.zip › G34C.tif]

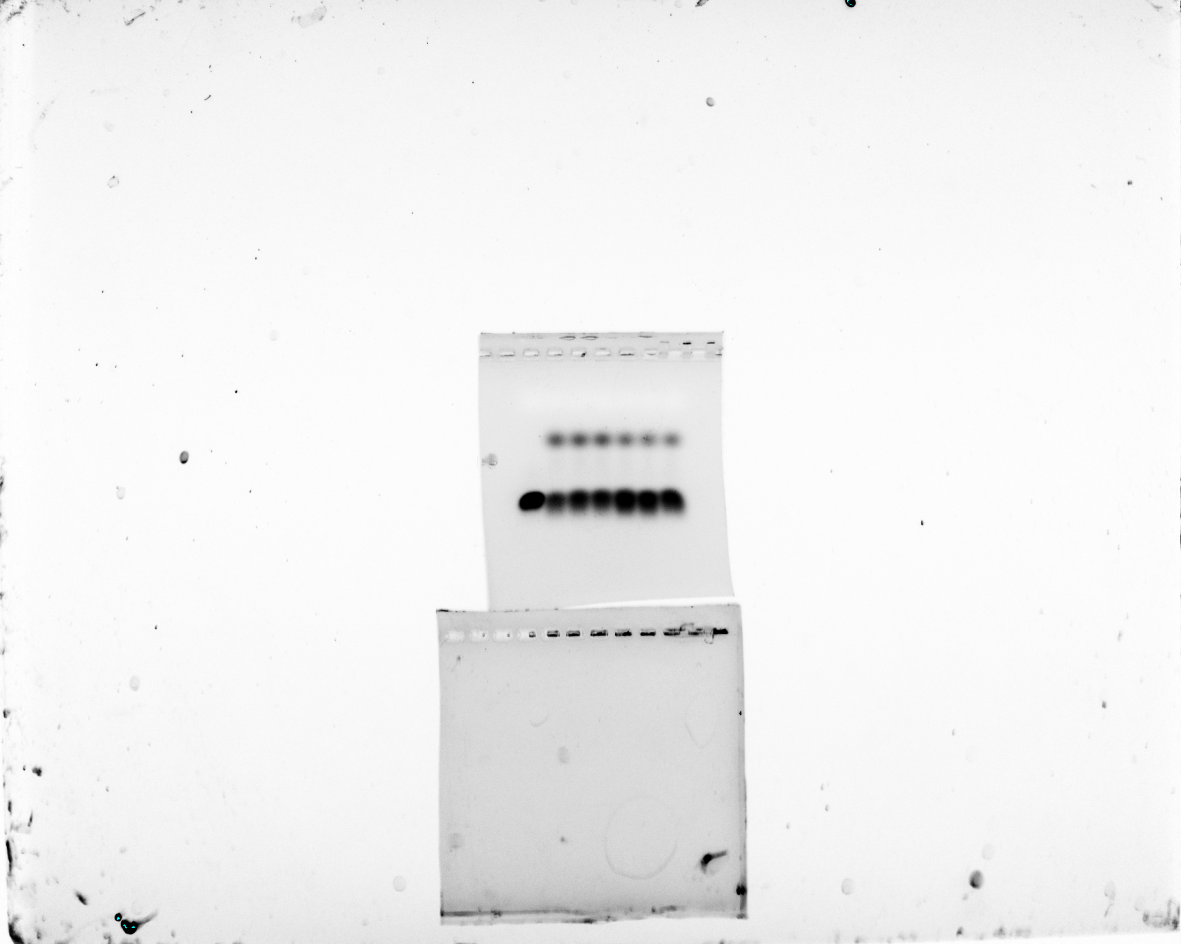

Supplement: Figure 5—source data 2. [file elife-107785-fig5-data2.zip › Ta.tif]

| RBD  |   |    | BSA  |   |    |
|------|---|----|------|---|----|
| Ta   |   | Tc | Ta   |   | Tc |
| G34C |   |    | G34C |   |    |
| -    | + | -  | +    | - | +  |

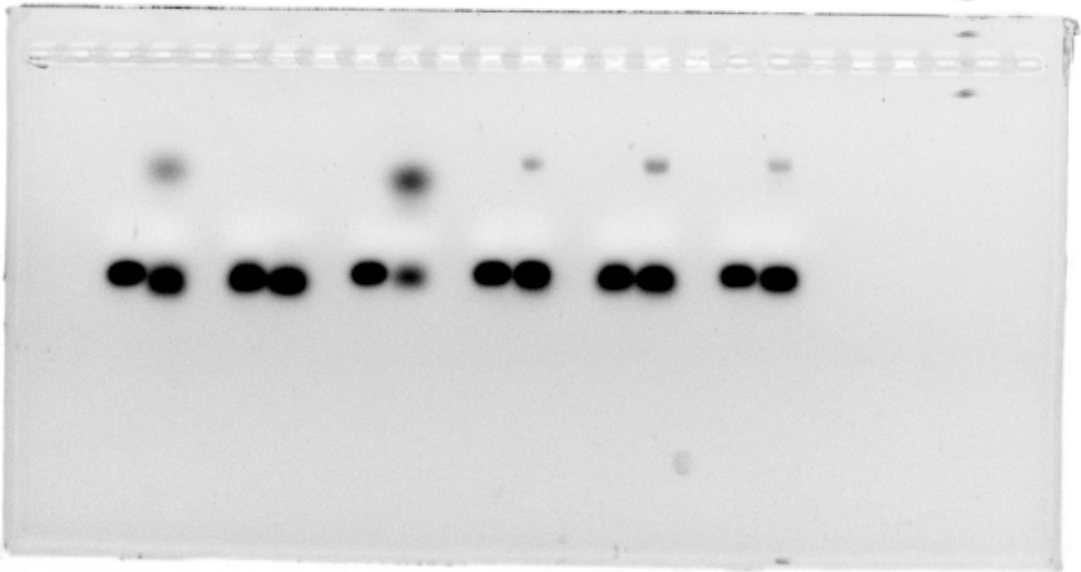

Replicate 1

| RBD  |   |    | BSA  |   |    |
|------|---|----|------|---|----|
| Ta   |   | Tc | Ta   |   | Tc |
| G34C |   |    | G34C |   |    |
| -    | + | -  | +    | - | +  |

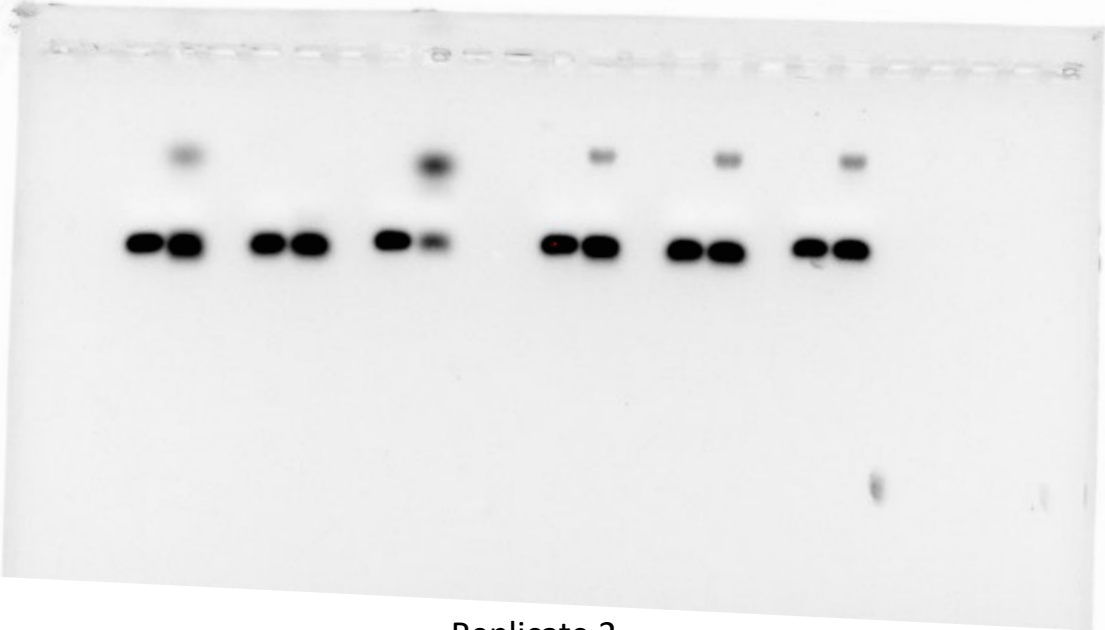

Replicate 2

Supplement: Figure 5—figure supplement 1—source data 1. [file elife-107785-fig5-figsupp1-data1.pdf]

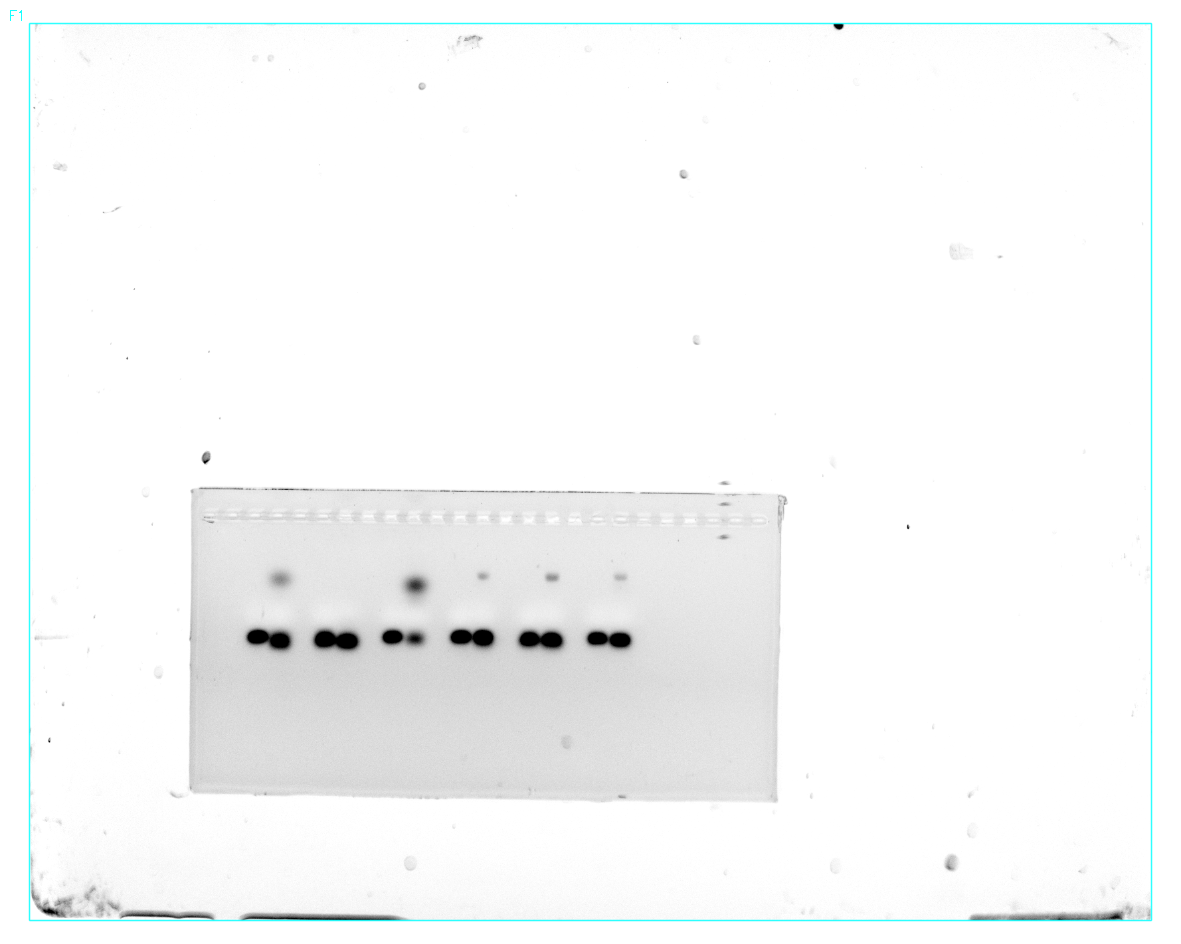

Supplement: Figure 5—figure supplement 1—source data 2. [file elife-107785-fig5-figsupp1-data2.zip › FigS10-Rep1.tif]

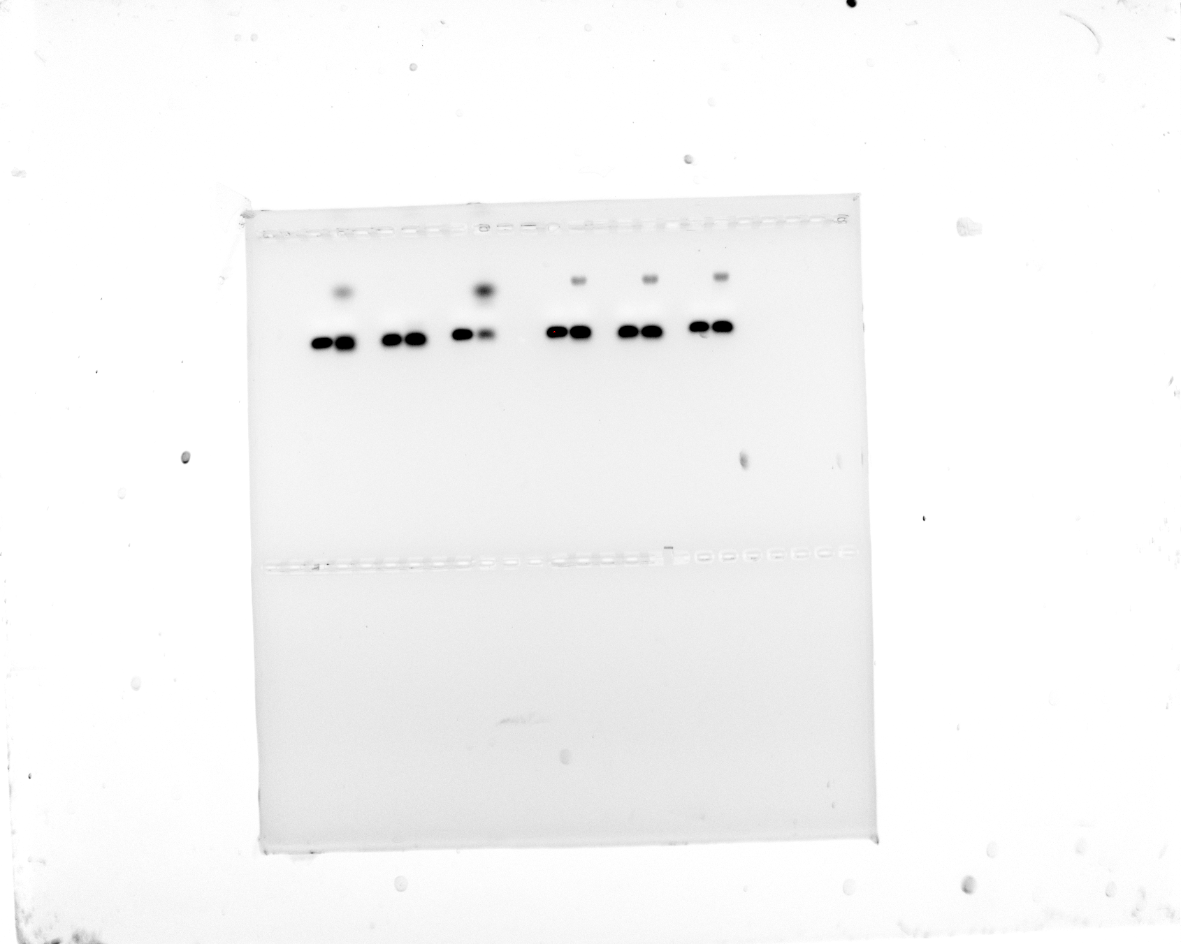

Supplement: Figure 5—figure supplement 1—source data 2. [file elife-107785-fig5-figsupp1-data2.zip › FigS10-Rep2.tif]

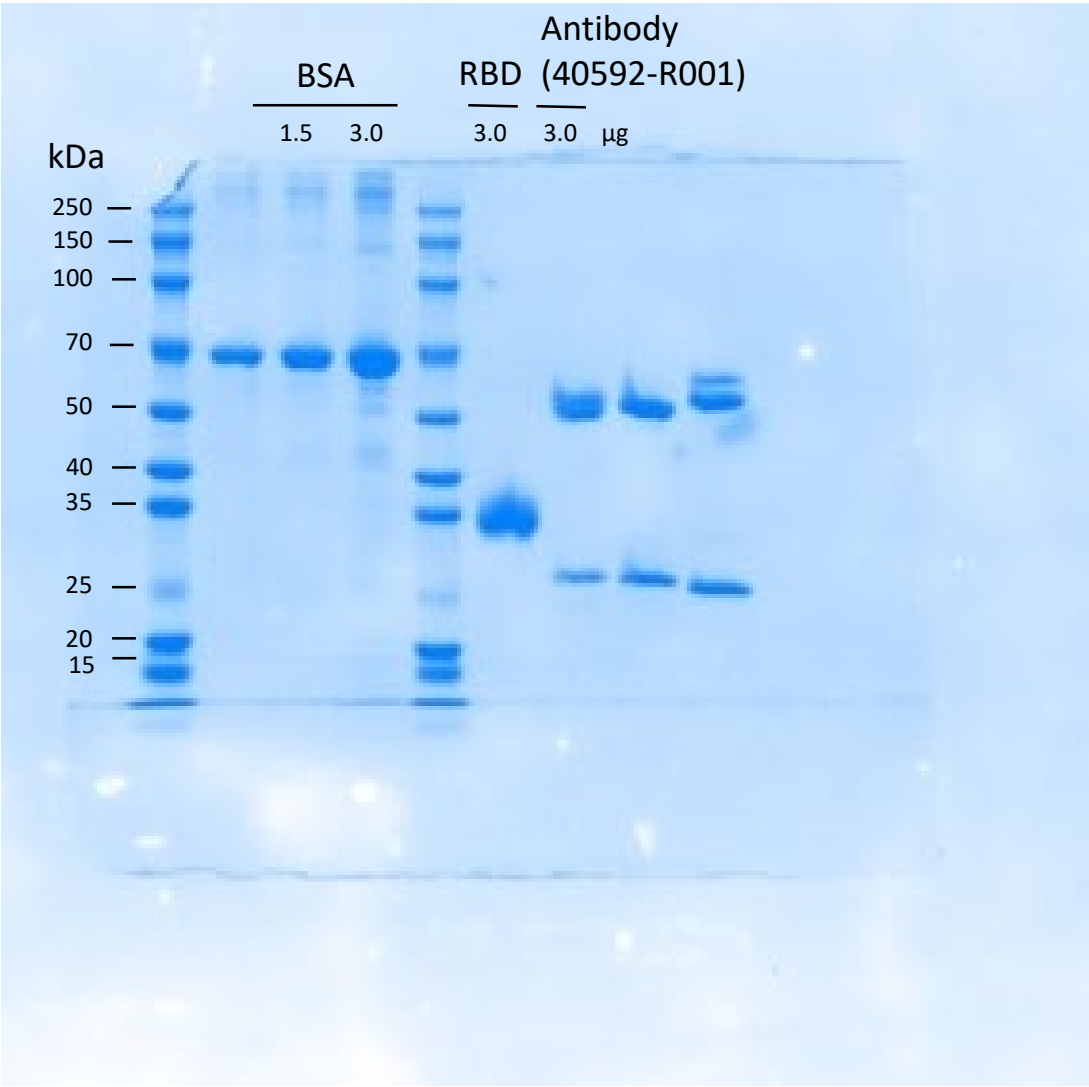

Supplement: Figure 5—figure supplement 2—source data 1. [file elife-107785-fig5-figsupp2-data1.pdf]

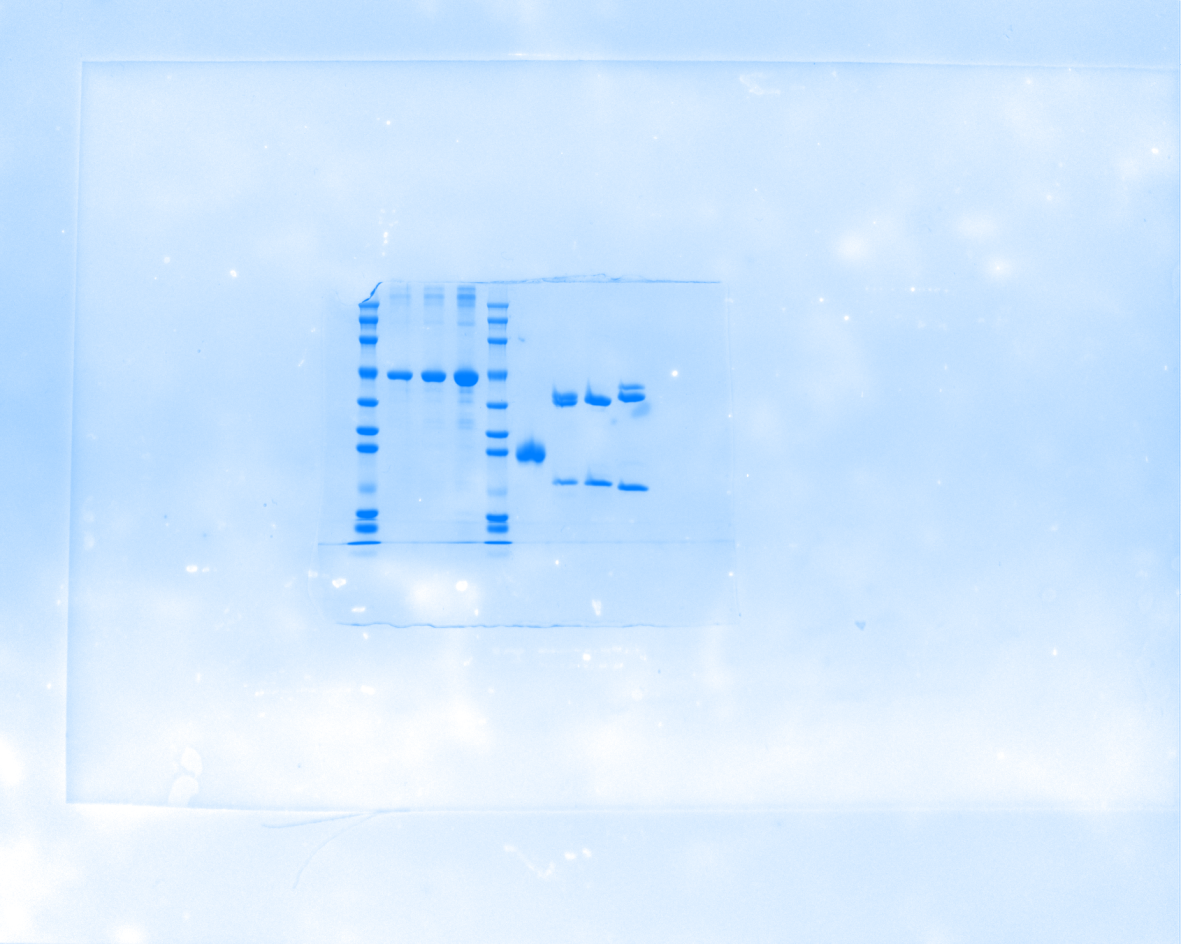

Supplement: Figure 5—figure supplement 2—source data 2. [file elife-107785-fig5-figsupp2-data2.zip › UNIVERSAL_11232023_150144_(Composite).tif]
