# Supplementary material for: In silico design and validation of high-affinity RNA aptamers for SARS-CoV-2 comparable to neutralizing antibodies
Source: eLife. 2026 Jul 14;14:RP107785. doi: 10.7554/eLife.107785 (PMC13368180; doi:10.7554/eLife.107785)
Supplement: Supplementary file 1. — (A) The relative binding free energy changes for single nucleotide mutations on Ta binding (mean ± standard deviation, kcal/mol, from five independent FEP runs). (B) The aptamer sequences employed in this study and their binding energies with RBD. (C) Relative binding free energy changes (ΔΔG, kcal/mol) for Ta binding to SARS-CoV-2 RBD variants (Alpha and Beta) calculated by FEP/HREX. (D) Relative binding free energy changes (ΔΔG, kcal/mol) for TaG34C binding to SARS-CoV-2 RBD variants calculated by FEP/HREX. (E) Summary of simulation systems in this study. [file elife-107785-supp1.docx]

**Supplementary Information**

**In silico design and validation of high-affinity RNA aptamers for SARS-CoV-2 comparable to neutralizing antibodies**

Yanqing Yang^1,4^, Lulu Qiao^2,4^, Yangwei Jiang^1^, Zhiye Wang^2, 3,*^, Dong Zhang^1,*^, Damiano Buratto^1^, Liquan Huang^1^, and Ruhong Zhou^1,3,*^

^1^Institute of Quantitative Biology, College of Life Sciences, Zhejiang University, Hangzhou, Zhejiang 310058, China.

^2^State Key Laboratory of Plant Environmental Resilience, College of Life Sciences, Zhejiang University, Hangzhou, Zhejiang 310058, China.

^3^The First Affiliated Hospital, College of Medicine, Zhejiang University, Hangzhou, Zhejiang 310058, China.

^4^These authors contributed equally: Yanqing Yang and Lulu Qiao.

* To whom correspondence should be addressed: [rhzhou@zju.edu.cn](mailto:rhzhou@zju.edu.cn) (R.Z.); [zhangd_iqb@zju.edu.cn](mailto:zhangd_iqb@zju.edu.cn) (D.Z.); [wangzhiye1@zju.edu.cn](mailto:wangzhiye1@zju.edu.cn) (Z.W.).

**Table A.** The relative binding free energy changes for single nucleotide mutations on Ta binding. Mean ± standard deviation (kcal/mol) from five independent FEP runs are given.

| **Mutation** | ***ΔG^bond^*** | ***ΔG^free^*** | ***ΔΔG^calc^*** |
| --- | --- | --- | --- |
| A14G | -53.12±0.27 | -53.22±0.06 | 0.10±0.28 |
| A14C | -81.05±0.11 | -82.58±0.07 | 1.54±0.13 |
| A14U | 18.99±0.19 | 18.31±0.05 | 0.68±0.20 |
| C23A | 84.01±0.20 | 86.16±0.38 | -2.15±0.43 |
| C23G | 30.35±0.24 | 32.84±0.42 | -2.49±0.49 |
| C23U | 102.20±0.45 | 104.16±0.37 | -1.96±0.58 |
| C24A | 82.30±0.18 | 82.61±0.16 | -0.30±0.24 |
| C24U | 99.57±0.43 | 99.11±0.18 | 0.47±0.47 |
| G25A | 57.94±0.37 | 56.48±0.34 | 1.47±0.50 |
| G25C | -22.95±0.41 | -24.89±0.14 | 1.94±0.43 |
| G25U | 78.16±0.24 | 76.26±0.25 | 1.90±0.35 |
| U27A | -18.65±0.04 | -19.19±0.09 | 0.54±0.10 |
| U27G | -70.85±0.09 | -71.51±0.13 | 0.66±0.16 |
| U27C | -99.46±0.18 | -99.78±0.08 | 0.32±0.20 |
| A28G | -52.81±0.13 | -52.97±0.12 | 0.16±0.17 |
| A28C | -82.67±0.06 | -82.92±0.05 | 0.26±0.08 |
| A28U | 17.93±0.04 | 17.55±0.05 | 0.37±0.06 |
| C29A | 89.42±0.30 | 83.08±0.28 | 6.34±0.41 |
| C29G | 34.47±0.75 | 29.91±0.13 | 4.66±0.76 |
| C29U | 100.81±0.11 | 98.91±0.16 | 1.89±0.28 |
| U30A | -20.35±0.12 | -20.35±0.05 | 0.00±0.13 |
| U30C | -99.91±0.19 | -100.39±0.18 | 0.48±0.26 |
| C33A | 81.44±0.46 | 82.41±0.17 | -0.97±0.49 |
| C33U | 99.98±0.48 | 98.26±0.30 | 1.72±0.56 |
| G34A | 55.91±0.15 | 58.41±0.24 | -2.50±0.28 |
| G34C | -27.51±0.14 | -24.46±0.21 | -3.05±0.26 |
| G34U | 74.17±0.24 | 76.82±0.22 | -2.65±0.33 |
| A40G | -55.18±0.41 | -54.82±0.21 | -0.36±0.46 |
| A40C | -81.47±0.17 | -81.31±0.20 | -0.16±0.26 |
| A40U | 19.69±0.08 | 20.12±0.17 | -0.44±0.19 |
| A42G | -53.07±0.49 | -52.85±0.26 | -0.22±0.56 |
| G43A | 52.94±0.26 | 52.30±0.15 | 0.65±0.30 |
| G43U | 75.93±0.11 | 75.57±0.27 | 0.37±0.30 |

**Table B.** The aptamer sequences employed in this study and their binding energies with RBD.

| **Aptamer Name** | **Sequences** | ***K_d_* (µM)** | ***ΔΔG_exp_* (kcal/mol)** | ***ΔΔG_FEP_***  **(kcal/mol)** |
| --- | --- | --- | --- | --- |
| Ta | 5’-GGCGACAUUU  GUAAUUCCUG  GACCGAUACU  UCCGUCAGGA  CAGAGGUUGCCA-3’ | 110.7 | -- | -- |
| Tc | 5’-GGUCCUGGAC  CGAUACUUCC  GUCAGGACCA-3’ | -- | -- | -- |
| Ta^G34C^ | 5’- GGCGACAUUU  GUAAUUCCUG  GACCGAUACU  UCCCUCAGGA  CAGAGGUUGCCA-3’ | 33.5±1.6 | -0.71±0.03 | -3.05±0.26 |
| Ta^G34U^ | 5’- GGCGACAUUU  GUAAUUCCUG  GACCGAUACU  UCCUUCAGGA  CAGAGGUUGCCA-3’ | 54.7±2.0 | -0.42±0.02 | -2.65±0.33 |
| Ta^G34A^ | 5’- GGCGACAUUU  GUAAUUCCUG  GACCGAUACU  UCCAUCAGGA  CAGAGGUUGCCA-3’ | 55.5±4.1 | -0.42±0.04 | -2.50±0.08 |
| Ta^C23G^ | 5’- GGCGACAUUU  GUAAUUCCUG  GAGCGAUACU  UCCGUCAGGA  CAGAGGUUGCCA-3’ | 167.9 | 0.25±0.00 | -2.49±0.49 |
| Ta^C23A^ | 5’-GGCGACAUUU  GUAAUUCCUG  GAACGAUACU  UCCGUCAGGA  CAGAGGUUGCCA-3’ | 55.7±9.8 | -0.42±0.10 | -2.15±0.43 |
| Ta^C23U^ | 5’- GGCGACAUUU  GUAAUUCCUG  GAUCGAUACU  UCCGUCAGGA  CAGAGGUUGCCA-3’ | 48.7±2.0 | -0.49±0.02 | -1.96±0.58 |

**Table C.** Relative binding free energy changes (*ΔΔG*, kcal/mol) for Ta binding to SARS-CoV-2 RBD variants (Alpha and Beta) calculated by FEP/HREX. Experimental values (*ΔΔG_exp_*) are taken from Liu et al., PNAS 2021, doi: 10.1073/pnas.2112942118. FEP-calculated results (*ΔΔG_FEP_*) represent the mean ± standard deviation from five independent simulations.

| **Variants** | **Mutations** | ***ΔΔG_exp_*** | ***ΔΔG_FEP_*** |
| --- | --- | --- | --- |
| 20I (Alpha, V1) (B.1.1.7) | **N501Y** | -0.24 | -0.42±0.07 |
| 20H (Beta, V2) (B.1.351) | **K417N, E484K, N501Y** | 0.36 | 0.64±0.25 |

**Table D.** Relative binding free energy changes (*ΔΔG*, kcal/mol) for Ta^G34C^ binding to SARS-CoV-2 RBD variants calculated by FEP/HREX. The “Mutations” column lists all amino acid mutations within the RBD region (residues 333–527). Mutations located within 5 Å of the aptamer are highlighted in red and were explicitly perturbed in the FEP calculations. Values represent the mean ± standard deviation from five independent runs.

| **Variants** | **Mutations** | ***ΔΔG_FEP_*** |
| --- | --- | --- |
| 20I (Alpha, V1) (B.1.1.7) | **N501Y** | -0.81±0.08 |
| 20H (Beta, V2) (B.1.351) | **K417N, E484K, N501Y** | -0.67±0.26 |
| 20J (Gamma, V3) (P.1) | **K417T, E484K, N501Y** | -0.72±0.25 |
| 21K (Omicron) (BA.1) | **G339D, S371L, S373P, S375F, K417N, N440K, G446S, S477N, T478K, E484A, Q493R, G496S, Q498R, N501Y, Y505H** | -3.00±0.52 |
| 21L (Omicron) (BA.2) | **G339D, S371F, S373P, S375F, T376A, D405N, R408S, K417N, N440K, S477N, T478K, E484A, Q493R, Q498R, N501Y, Y505H** | -2.54±0.60 |
| 22A (Omicron) (BA.4) | **G339D, S371F, S373P, S375F, T376A, D405N, R408S, K417N, N440K, L452R, S477N, T478K, E484A, F486V, Q498R, N501Y, Y505H** | 2.11±0.67 |
| 22B (Omicron) (BA.5) | **G339D, S371F, S373P, S375F, T376A, D405N, R408S, K417N, N440K, L452R, S477N, T478K, E484A, F486V, Q498R, N501Y, Y505H** | 2.27±0.68 |
| 22C (Omicron) (BA.2.12.1) | **G339D, S371F, S373P, S375F, T376A, D405N, R408S, K417N, N440K, L452Q, S477N, T478K, E484A, Q493R, Q498R, N501Y, Y505H** | -2.40±0.41 |
| 22D (Omicron) (BA.2.75) | **G339H, S371F, S373P, S375F, T376A, D405N, R408S, K417N, N440K, G446S, N460K, S477N, T478K, E484A, Q493R, Q498R, N501Y, Y505H** | -5.03±0.81 |
| 22E (Omicron) (BQ.1) | **G339D, S371F, S373P, S375F, T376A, D405N, R408S, K417N, N440K, K444T, L452R, N460K, S477N, T478K, E484A, F486V, Q493R, Q498R, N501Y, Y505H** | -0.26±0.75 |
| 22F (Omicron) (XBB) | **G339H, R346T, L368I, S371F, S373P, S375F, T376A, D405N, R408S, K417N, N440K, V445P, G446S, N460K, S477N, T478K, E484A, F486S, F490S, Q493R, Q498R, N501Y, Y505H** | -3.13±0.73 |
| 23A (Omicron) (XBB.1.5) | **G339H, R346T, L368I, S371F, S373P, S375F, T376A, D405N, R408S, K417N, N440K, V445P, G446S, N460K, S477N, T478K, E484A, F486P, F490S, Q493R, Q498R, N501Y, Y505H** | -2.28±0.96 |
| 23B (Omicron) (XBB.1.16) | **G339H, R346T, L368I, S371F, S373P, S375F, T376A, D405N, R408S, K417N, N440K, V445P, G446S, N460K, S477N, T478R, E484A, F486P, F490S, Q493R, Q498R, N501Y, Y505H** | -2.64±0.79 |
| 23C (Omicron) (CH.1.1) | **G339H, R346T, S371F, S373P, S375F, T376A, D405N, R408S, K417N, N440K, K444T, G446S, L452R, N460K, S477N, T478K, E484A, F486S, Q493R, Q498R, N501Y, Y505H** | -0.80±0.95 |
| 23D (Omicron) (XBB.1.9) | **G339H, R346T, L368I, S371F, S373P, S375F, T376A, D405N, R408S, K417N, N440K, V445P, G446S, N460K, S477N, T478K, E484A, F486S, F490S, Q493R, Q498R, N501Y, Y505H** | -4.34±0.68 |
| 23E (Omicron) (XBB.2.3) | **G339H, R346T, L368I, S371F, S373P, S375F, T376A, D405N, R408S, K417N, N440K, V445P, G446S, N460K, S477N, T478K, E484A, F486P, F490S, Q493R, Q498R, N501Y, Y505H** | -3.28±1.06 |
| 23F (Omicron) (EG.5.1) | **G339H, R346T, L368I, S371F, S373P, S375F, T376A, D405N, R408S, K417N, N440K, V445P, G446S, F456L, N460K, S477N, T478K, E484A, F486P, F490S, Q493R, Q498R, N501Y, Y505H** | -5.32±1.38 |
| 23G (Omicron) (XBB.1.5.70) | **G339H, R346T, L368I, S371F, S373P, S375F, T376A, D405N, R408S, K417N, N440K, V445P, G446S, L455F, F456L, N460K, S477N, T478K, E484A, F486P, F490S, Q493R, Q498R, N501Y, Y505H** | -4.23±0.77 |
| 23H (Omicron) (HK.3) | **G339H, R346T, L368I, S371F, S373P, S375F, T376A, D405N, R408S, K417N, N440K, V445P, G446S, L455F, F456L, N460K, S477N, T478K, E484A, F486P, F490S, Q493R, Q498R, N501Y, Y505H** | -4.63±0.96 |
| 23I (Omicron) (BA.2.86) | **G339H, K356T, S371F, S373P, S375F, T376A, R403K, D405N, R408S, K417N, N440K, V445H, G446S, N450D, L452W, N460K, S477N, T478K, N481K, E484K, F486P, Q493R, Q498R, N501Y, Y505H** | -1.89±1.27 |
| 24A (Omicron) (JN.1) | **G339H, K356T, S371F, S373P, S375F, T376A, R403K, D405N, R408S, K417N, N440K, V445H, G446S, N450D, L452W, L455S, N460K, S477N, T478K, N481K, E484K, F486P, Q493R, Q498R, N501Y, Y505H** | -7.58±0.86 |
| 24B (Omicron) (JN.1.11.1) | **G339H, K356T, S371F, S373P, S375F, T376A, R403K, D405N, R408S, K417N, N440K, V445H, G446S, N450D, L452W, L455S, F456L, N460K, S477N, T478K, N481K, E484K, F486P, Q493R, Q498R, N501Y, Y505H** | -4.66±1.45 |
| 24C (Omicron) (KP.3) | **G339H, K356T, S371F, S373P, S375F, T376A, R403K, D405N, R408S, K417N, N440K, V445H, G446S, N450D, L452W, L455S, F456L, N460K, S477N, T478K, N481K, E484K, F486P, Q493E, Q498R, N501Y, Y505H** | 3.48±1.14 |

**Table E.** Summary of simulation systems in this study.

| **Name** | **Number of**  **atoms** | **Production simulation time** | **Description** |
| --- | --- | --- | --- |
| Ta (3D) | 1106 | 50 ns×10 replicas replica exchange MD, 2 runs | Predict RNA 3D structure in the coarse-grained IsRNA2 model for Ta aptamer |
| Tc (3D) | 636 | 50 ns×10 replicas replica exchange MD, 2 runs | Predict RNA 3D structure in the coarse-grained IsRNA2 model for Tc aptamer |
| RBD | 62442 | 500ns, 3 runs | Relax the 3D structure of RBD in all-atom MD simulations to prepare docking structure |
| RBD&Ta  (Conf 01) | 130136 | 500ns, 3 runs | Relax the 3D binding complex of RBD & Ta from initial conformation |
| RBD&Ta  (Conf 02) | 130136 | 500ns, 3 runs | Relax the 3D binding complex of RBD & Ta from initial conformation |
| RBD&Ta  (Conf 03) | 285645 | 500ns, 3 runs | Relax the 3D binding complex of RBD & Ta from initial conformation |
| RBD&Ta  (Conf 04) | 217783 | 500ns, 3 runs | Relax the 3D binding complex of RBD & Ta from initial conformation |
| RBD&Ta  (Conf 05) | 179307 | 500ns, 3 runs | Relax the 3D binding complex of RBD & Ta from initial conformation |
| RBD&Ta  (Conf 06) | 120009 | 500ns, 3 runs | Relax the 3D binding complex of RBD & Ta from initial conformation |
| RBD&Ta  (Conf 01)  (MM/GBSA) | 130136 | 100ns, 3 runs | Simulate the binding complex of Ta and RBD to perform MM/GBSA |
| RBD&Ta  (Conf 02)  (MM/GBSA) | 130136 | 100ns, 3 runs | Simulate the binding complex of Ta and RBD to perform MM/GBSA |
| RBD&Ta  (Conf 03)  (MM/GBSA) | 285645 | 100ns, 3 runs | Simulate the binding complex of Ta and RBD to perform MM/GBSA |
| RBD&Ta  (Conf 04)  (MM/GBSA) | 217783 | 100ns, 3 runs | Simulate the binding complex of Ta and RBD to perform MM/GBSA |
| RBD&Ta  (Conf 05)  (MM/GBSA) | 179307 | 100ns, 3 runs | Simulate the binding complex of Ta and RBD to perform MM/GBSA |
| RBD&Ta  (Conf 06)  (MM/GBSA) | 120009 | 100ns, 3 runs | Simulate the binding complex of Ta and RBD to perform MM/GBSA |
| RBD&Ta  (Conf 01)  (SMD) | 284617 | ~50ns, 4 runs | Separate the bound Ta from the RBD to perform SMD |
| RBD&Ta  (Conf 02)  (SMD) | 130136 | ~50ns, 4 runs | Separate the bound Ta from the RBD to perform SMD |
| RBD&Ta  (Conf 03)  (SMD) | 285645 | ~50ns, 4 runs | Separate the bound Ta from the RBD to perform SMD |
| RBD&Ta  (Conf 04)  (SMD) | 217783 | ~50ns, 4 runs | Separate the bound Ta from the RBD to perform SMD |
| RBD&Tc  (Conf 01) | 60549 | 500ns, 3 runs | Relax the 3D binding complex of RBD & Tc from initial conformation |
| RBD&Tc  (Conf 02) | 51210 | 500ns, 3 runs | Relax the 3D binding complex of RBD & Tc from initial conformation |
| RBD&Tc  (Conf 03) | 47448 | 500ns, 3 runs | Relax the 3D binding complex of RBD & Tc from initial conformation |
| RBD&Tc  (Conf 04) | 50101 | 500ns, 3 runs | Relax the 3D binding complex of RBD & Tc from initial conformation |
| RBD&Tc  (Conf 01)  (MM/GBSA) | 60549 | 100ns, 3 runs | Simulate the binding complex of Tc and RBD to perform MM/GBSA |
| RBD&Tc  (Conf 02)  (MM/GBSA) | 51210 | 100ns, 3 runs | Simulate the binding complex of Tc and RBD to perform MM/GBSA |
| RBD&Tc  (Conf 03)  (MM/GBSA) | 47448 | 100ns, 3 runs | Simulate the binding complex of Tc and RBD to perform MM/GBSA |
| RBD&Tc  (Conf 04)  (MM/GBSA) | 50101 | 100ns, 3 runs | Simulate the binding complex of Tc and RBD to perform MM/GBSA |
| RBD&Tc  (Conf 01)  (SMD) | 60549 | ~50ns, 4 runs | Separate the bound Tc from the RBD to perform SMD |
| RBD&Tc  (Conf 02)  (SMD) | 51210 | ~50ns, 4 runs | Separate the bound Tc from the RBD to perform SMD |
| RBD&Tc  (Conf 03)  (SMD) | 47448 | ~50ns, 4 runs | Separate the bound Tc from the RBD to perform SMD |
| RBD&Tc  (Conf 04)  (SMD) | 50101 | ~50ns, 4 runs | Separate the bound Tc from the RBD to perform SMD |
| RBD&ACE2 | 117622 | 500ns, 3 runs | Simulate the 3D binding complex of RBD & ACE2 to calculate contact ratios |
| RBD&P2C-1F11 | 254496 | 500ns, 3 runs | Relax the 3D binding complex of RBD & P2C-1F11 from initial conformation |
| RBD&2H2 Fab | 364742 | 500ns, 3 runs | Relax the 3D binding complex of RBD & 2H2 Fab from initial conformation |
| RBD&S2E12 Fab | 291645 | 500ns, 3 runs | Relax the 3D binding complex of RBD & S2E12 Fab from initial conformation |
| RBD&ACE2  (MM/GBSA) | 117622 | 100ns, 3 runs | Simulate the binding complex of ACE2 and RBD to perform MM/GBSA |
| RBD&P2C-1F11  (MM/GBSA) | 254496 | 100ns, 3 runs | Simulate the binding complex of P2C-1F11 and RBD to perform MM/GBSA |
| RBD&2H2 Fab  (MM/GBSA) | 364742 | 100ns, 3 runs | Simulate the binding complex of 2H2 Fab and RBD to perform MM/GBSA |
| RBD&S2E12 Fab  (MM/GBSA) | 291645 | 100ns, 3 runs | Simulate the binding complex of S2E12 Fab and RBD to perform MM/GBSA |
| Ta (free) | 129115 | 500ns, 3 runs | Relax the 3D structure of Ta in all-atom MD simulations to prepare FEP free state |
| RBD&Ta^G34C^ | 108541 | 500ns, 3 runs | Simulate the binding complex of Ta^G34C^ mutation & RBD |
