# Supplementary material for: In silico design and validation of high-affinity RNA aptamers for SARS-CoV-2 comparable to neutralizing antibodies
Source: eLife. 2026 Jul 14;14:RP107785. doi: 10.7554/eLife.107785 (PMC13368180; doi:10.7554/eLife.107785)
Supplement: Peer review history

# Editor report: eLife Assessment

**DOI:** 10.7554/eLife.107785.4.sa0

**Authors:** Aaron Frank

**Affiliations:** Arrakis Therapeutics United States

**Keywords:** Solid, Valuable

This **valuable** study introduces CAAMO, a computational framework that combines structure prediction, in silico mutagenesis, molecular simulations, and energy calculations to design RNA aptamers with improved binding affinity. The computational methodology is **solid**, demonstrating strong theoretical foundations and systematic integration of multiple prediction techniques. Many of the previously identified methodological weaknesses that limit the strength of support for the computational predictions have been addressed.

# Referee report: Reviewer #4 (Public review)

**DOI:** 10.7554/eLife.107785.4.sa1

[Editors' note: this version has been assessed by the Reviewing Editor without further input from the original reviewers. The authors have addressed the comments raised in the previous round of review.]

Summary:

The authors demonstrate a computational rational design approach for developing RNA aptamers with improved binding to the Receptor Binding Domain (RBD) of the SARS-CoV-2 spike protein. They demonstrate the ability of their approach to improve binding affinity using a previously identified RNA aptamer, RBD-PB6-Ta, which binds to the RBD. They also computationally estimate the binding energies of various RNA aptamers with the RBD and compare against RBD binding energies for a few neutralizing antibodies from the literature. Finally, experimental binding affinities are estimated by electrophoretic mobility shift assays (EMSA) for various RNA aptamers and a single commercially available neutralizing antibody to support the conclusions from computational studies on binding. The authors conclude that their computational framework, CAAMO, can provide reliable structure predictions and effectively support rational design of improved affinity for RNA aptamers towards target proteins. Additionally, they claim that their approach achieved design of high affinity RNA aptamer variants that bind to the RBD as well or better than a commercially available neutralizing antibody.

Strengths:

The thorough computational approaches employed in the study provide solid evidence of the value of their approach for computational design of high affinity RNA aptamers. The theoretical analysis using Free Energy Perturbation (FEP) to estimate relative binding energies supports the claimed improvement of affinity for RNA aptamers and provides valuable insight into the binding model for the tested RNA aptamers in comparison to previously studied neutralizing antibodies. The multimodal structure prediction in the early stages of the presented CAAMO framework, combined with the demonstrated outcome of improved affinity using the structural predictions as a starting point for rational design, provide moderate confidence in the structure predictions.

# Author comment: Author response

**DOI:** 10.7554/eLife.107785.4.sa2

**Authors:** Yanqing Yang, Lulu Qiao, Yangwei Jiang, Zhiye Wang, Dong Zhang, Damiano Buratto, Liquan Huang, Ruhong Zhou

**Affiliations:** Zhejiang University Hangzhou China; Zhejiang University HangZhou China; Zhejiang University Hangzhou China; Zhejiang University Hangzhou China; Zhejiang University Hangzhou China; Zhejiang University Hangzhou China; Zhejiang University Hangzhou China; Zhejiang University Hangzhou China

The following is the authors’ response to the previous reviews

**Reviewer #4 (Public review):**

Summary:

The authors demonstrate a computational rational design approach for developing RNA aptamers with improved binding to the Receptor Binding Domain (RBD) of the SARS-CoV-2 spike protein. They demonstrate the ability of their approach to improve binding affinity using a previously identified RNA aptamer, RBD-PB6-Ta, which binds to the RBD. They also computationally estimate the binding energies of various RNA aptamers with the RBD and compare against RBD binding energies for a few neutralizing antibodies from the literature. Finally, experimental binding affinities are estimated by electrophoretic mobility shift assays (EMSA) for various RNA aptamers and a single commercially available neutralizing antibody to support the conclusions from computational studies on binding. The authors conclude that their computational framework, CAAMO, can provide reliable structure predictions and effectively support rational design of improved affinity for RNA aptamers towards target proteins. Additionally, they claim that their approach achieved design of high affinity RNA aptamer variants that bind to the RBD as well or better than a commercially available neutralizing antibody.

Strengths:

The thorough computational approaches employed in the study provide solid evidence of the value of their approach for computational design of high affinity RNA aptamers. The theoretical analysis using Free Energy Perturbation (FEP) to estimate relative binding energies supports the claimed improvement of affinity for RNA aptamers and provides valuable insight into the binding model for the tested RNA aptamers in comparison to previously studied neutralizing antibodies. The multimodal structure prediction in the early stages of the presented CAAMO framework, combined with the demonstrated outcome of improved affinity using the structural predictions as a starting point for rational design, provide moderate confidence in the structure predictions.

We thank the reviewer for this accurate summary and for recognizing the strength of our integrated computational–experimental workflow in improving aptamer affinity.

Weaknesses:

The experimental characterization of RBD affinities for the antibody and RNA aptamers in this study present serious concerns regarding the methods used and the data presented in the manuscript, which call into question the major conclusions regarding affinity towards the RBD for their aptamers compared to antibodies. The claim that structural predictions from CAAMO are reasonable is rational, but this claim would be significantly strengthened by experimental validation of the structure (i.e. by chemical footprinting or solving the RBD-aptamer complex structure).

The conclusions in this work are somewhat supported by the data, but there are significant issues with experimental methods that limit the strength of the study's conclusions.

(1) The EMSA experiments have a number of flaws that limit their interpretability. The uncropped electrophoresis images, which should include molecular size markers and/or positive and negative controls for bound and unbound complex components to support interpretation of mobility shifts, are not presented. In fact, a spliced image can be seen for Figure 4E, which limits interpretation without the full uncropped image.

Thank you for your valuable comments and careful review.

In response to your suggestion, we have now provided all uncropped electrophoresis raw images corresponding to the results in the main figures and supplementary figures (Fig. 2F, 3D, 3E, 4E, S9A, S10 and S11 of the original manuscript) in the revised version. Regarding the spliced image in Fig. 4E, the uncropped raw gel image clearly shows that the two C23U samples were run on an adjacent lane of the same gel due to the total number of samples exceeding the well capacity of a single lane. All samples were electrophoresed and signal-detected under identical experimental conditions in one single experiment, ensuring the validity of direct signal intensity comparison across all samples. These complete uncropped raw images have been supplemented in the revised manuscript as Fig. S12.

The following highlighted words have been added to the revised manuscript.

“All uncropped raw gel images corresponding to these EMSA experiments are provided in Supplementary Fig. S12.”

Additionally, the volumes of EMSA mixtures are not presented when a mass is stated (i.e. for the methods used to create Figure 3D), which leaves the reader without the critical parameter, molar concentration, and therefore leaves in question the claim that the tested antibody is high affinity under the tested conditions.

Thank you for your valuable comment on this oversight.

For the EMSA assay in Fig. 3D, the reaction mixture (10 μL total volume) contained 3 μg of RBD protein and 3 μg of antibody (40592-R001), either individually or in combination, with incubation at room temperature for 20 minutes. Based on the molecular weights (35 kDa for RBD and 150 kDa for the IgG antibody), the corresponding molar concentrations in the mixture were calculated as 8.57 μM for RBD and 2 μM for the antibody. To ensure consistency, clarity and provide the critical molar concentration parameter, we have revised the legend of Fig. 3D, replacing the mass values with the calculated molar concentrations as you suggested.

The following highlighted words have been added to the revised manuscript.

“(D) Binding ability of the commercial antibody (40592-R001) to RBD was assessed by native-PAGE. The reaction mixture (10 μL) contained 8.57 μM RBD protein and 2 μM antibody, incubated individually or combined, followed by Coomassie brilliant blue staining.”

Additionally, protein should be visualized in all gels as a control to ensure that lack of shifts is not due to absence/aggregation/degradation of the RBD protein. In the case of Figure 3E, for example, it can be seen that there are degradation products included in the RBD-only lane, introducing a reasonable doubt that the lack of a shift in RNA tests (i.e. Figure 2F) is conclusively due to a lack of binding.

We sincerely appreciate your careful evaluation of our work, which helps us further clarify the experimental details and data reliability.

First, we would like to clarify the nature of the gel electrophoresis in Fig. 3E: the RBD protein was separated by native-PAGE rather than denaturing SDS-PAGE. The RBD protein used in all experiments was purchased from HUABIO (Cat. No. HA210064) with guaranteed quality, and its integrity and purity were independently verified in our laboratory via denaturing SDS-PAGE (see revised Fig. S11), which showed a single, intact band without any degradation products. The ladder-like bands observed in the RBD-only lane of the native-PAGE gel are not a result of protein degradation. Instead, they arise from two well-characterized properties of recombinant SARS-CoV-2 Spike RBD protein expressed in human cells: intrinsic conformational heterogeneity (the RBD domain exists in multiple dynamic conformations due to its structural flexibility) (Cai et al., Science, 2020; Wrapp et al., Science, 2020) and heterogeneity in N-glycosylation modification (variable glycosylation patterns at the conserved N-glycosylation sites of RBD) (Casalino et al., ACS Cent. Sci., 2020; Ives et al., eLife, 2024), both of which could cause distinct migration bands in native-PAGE under non-denaturing conditions.

Second, to ensure the reliability of the RNA-binding results, the EMSA experiments for determining the binding affinity (K_d_) of RBD to Ta, Tc and Ta variants were performed with three independent biological replicates (the original manuscript includes all replicate data in Fig. 2F and S9). Consistent results were obtained across all replicates, which effectively rules out false-negative outcomes caused by accidental absence or loss of functional RBD protein in the reaction system. In addition, our gel images (Fig. 2F and S9 in original manuscript) and uncropped raw images of all EMSA gels (Fig. S12 in revised manuscript) show no significant signal accumulation in the sample wells, confirming the absence of RBD protein aggregation in the binding reactions—an issue that would otherwise interfere with RNA-protein interaction and band shift detection.

New results for RBD analysis by denaturing SDS-PAGE, along with the associated discussion, have been added to the revised manuscript (Fig. S11).

References

Cai, Y. et al. Distinct conformational states of SARS-CoV-2 spike proteins. Science 369, 1586-1592 (2020).

Casalino, L. et al. Beyond shielding: the roles of glycans in the SARS-CoV-2 spike protein. ACS Cent. Sci. 6, 1722-1734 (2020).

Ives, C.M. et al. Role of N343 glycosylation on the SARS-CoV-2 S RBD structure and co-receptor binding across variants of concern. eLife 13, RP95708 (2024).

Wrapp, D. et al. Cryo-EM structure of the 2019-nCoV spike in the prefusion conformation. Science 367, 1260-1263 (2020).

The following highlighted words have been added to the revised manuscript.

“The integrity and purity of the RBD protein were confirmed by denaturing SDS-PAGE (Fig. S11), showing a single intact band without degradation. The multiple bands observed in native PAGE (e.g., Fig. 3E) are due to conformational and glycosylation heterogeneity [63–66] rather than protein degradation. To rule out non-specific aptamer–protein interactions, BSA was additionally included as a non-target protein control in EMSA assays; the wild-type Ta, the negative control Tc, and the optimized Ta^G34C^ all showed only weak, comparable background signals with BSA but distinct target-specific binding to RBD (Fig. S10). Uncropped EMSA gel images (Fig. S12) and consistent results from three biological replicates (Fig. 2F and S9) confirm the absence of protein aggregation and ensure data reliability.”

Finally, there is no control for nonspecific binding, such as BSA or another non-target protein, which fails to eliminate the possibility of nonspecific interactions between their designed aptamers and proteins in general. A nonspecific binding control should be included in all EMSA experiments.

Thank you for this constructive comment.

Following your recommendation, we have supplemented the EMSA assays with BSA as a non-target protein control to rule out non-specific binding between our designed aptamers (Ta, Tc and Ta^G34C^) and exogenous proteins. The results revealed that all three aptamers (Ta, Tc and Ta^G34C^) exhibited only weak and comparable background signals with BSA (Fig. S10), which may originate from BSA itself or trace contaminating proteins in the protein sample (Fig. S11). The similar intensities of these background signals across Ta, Tc, and Ta^G34C^ indicate a comparable, low level of non-specific binding among these aptamers (Fig. S10). In sharp contrast, RBD displayed markedly stronger binding toward Ta^G34C^ than Ta, while no detectable binding was observed with the negative control Tc (Fig. S10). Collectively, these results verify that the aptamer–RBD interactions characterized in this study are target-specific and exclude non-specific aptamer–protein interactions.

All the new experimental data of the non-specific binding controls have been integrated into the revised manuscript (Fig. S10) and the corresponding results and Methods have been updated accordingly. The following highlighted words have been added to the revised manuscript:

“To further exclude non-specific aptamer–protein interactions, we performed parallel EMSA assays using bovine serum albumin (BSA) as a non-target protein control for Ta, Tc, and the optimized Ta^G34C^ (see Fig. S10). Only weak, comparable background signals were observed for all three aptamers with BSA. Such minor non-specific binding may originate from BSA itself or trace contaminating proteins in the BSA samples (Fig. S10). In contrast, markedly stronger binding was detected between RBD and Ta or Ta^G34C^, whereas no detectable binding was observed with the negative control Tc (Figs. 4E, S10). Such distinct binding profiles of aptamers with RBD and BSA confirm that the aptamer–RBD interactions characterized in this study are target-specific.”

“To rule out non-specific aptamer–protein interactions, BSA was additionally included as a non-target protein control in EMSA assays; the wild-type Ta, the negative control Tc, and the optimized Ta^G34C^ all showed only weak, comparable background signals with BSA but distinct target-specific binding to RBD (Fig. S10).”

(2) The evidence supporting claims of better binding to RBD by the aptamer compared to the commercial antibody is flawed at best. The commercial antibody product page indicates an affinity in low nanomolar range, whereas the fitted values they found for the aptamers in their study are orders of magnitude higher at tens of micromolar. Moreover, the methods section is lacking in the details required to appropriately interpret the competitive binding experiments. With a relatively short 20-minute equilibration time, the order of when the aptamer is added versus the antibody makes a difference in which is apparently bound. The issue with this becomes apparent with the lack of internal consistency in the presented results, namely in comparing Fig 3E (which shows no interference of Ta binding with 5uM antibody) and Fig 5D (which shows interference of Ta binding with 0.67-1.67uM antibody). The discrepancy between these figures calls into question the methods used, and it necessitates more details regarding experimental methods used in this manuscript.

Thank you for your insightful comments, which have helped us refine the rigor of our study. We address each of your concerns in detail below:

First, we agree with your observation that the commercial neutralizing antibody (Sino Biological, Cat# 40592-R001) is reported to bind Spike RBD with low nanomolar affinity on its product page. However, this discrepancy in affinity values (nanomolar vs. micromolar) stems from the use of distinct analytical methods. The product page affinity was determined via the Octet RED System, a technique analogous to Surface Plasmon Resonance (SPR) that offers high sensitivity for kinetic and affinity measurements. In contrast, our study employed EMSA, a method primarily optimized for semi-quantitative assessment of binding interactions. The inherent differences in sensitivity and principle between these two techniques—with Octet RED System enabling real-time monitoring of biomolecular interactions and EMSA relying on gel separation—account for the observed variation in affinity values.

Second, regarding the competitive binding experiments, we appreciate your note on the critical role of reagent addition order and equilibration time. To eliminate potential biases from sequential addition, we clarify that Cy3-labeled RNAs, RBD proteins, and the neutralizing antibody were added simultaneously to the reaction system. We have revised the Methods section to provide a detailed protocol for the EMSA experiments, to ensure full reproducibility and appropriate interpretation of the results.

Third, we acknowledge and apologize for a critical error in the figure legends of Fig. 3E: the concentrations reported (5 μM aptamer and antibody 40592-R001) refer to stock solutions, not the final concentrations in the EMSA reaction mixture. The correct final concentrations are 0.5 μM for aptamer Ta, and 0.5 μM for the antibody. This correction resolves the apparent inconsistency between Fig. 3E and Fig. 5D, as the final antibody concentration in Fig. 3E is now consistent with the concentration range used in Fig. 5D. We have updated the figure legends for Fig. 3E and revised the Methods section to explicitly distinguish between stock and final reaction concentrations, ensuring clarity and internal consistency of the results.

We sincerely thank you for highlighting these issues, which have prompted important revisions to improve the clarity, accuracy, and rigor of our manuscript.

The following highlighted words have been added to the revised manuscript.

“For competitive binding experiments, Cy3-labelled RNAs, RBD proteins, and neutralizing antibody 40592-R001 were added simultaneously to the EMSA buffer and incubated at room temperature for 20 min.”

“(E) The RBD binding abilities of the aptamer Ta and commercial antibody 40592-R001 were compared by EMSA competitive binding experiments. The aptamer-RBD complex bands were shown after running on an agarose gel following the incubation of 40 μM RBD protein, 0.5 μM aptamer Ta, and 0.5 μM antibody 40592-R001 (final concentrations in the reaction mixture).”

“(D) EMSA images of competitive binding experiments to characterize the RBD binding abilities of RNA aptamers (WT Ta and Ta^G34C^) and the commercial monoclonal SARS-CoV-2 neutralizing antibody 40592-R001. The aptamer-RBD complex bands were showed by running an agarose gel after incubation of 40 μM of RBD protein and 0.5 μM indicated aptamer with varying concentrations of the antibody 40592-R001. Final antibody concentrations ranged from 0 to 1.67 μM in the reaction mixtures. Results showed that Ta^G34C^, but not WT Ta, exhibited a higher binding affinity to the RBD proteins than that of the antibody.”

(3) The utility of the approach for increasing affinity of RNA aptamers for their targets is well supported through computational and experimental techniques demonstrating relative improvements in binding affinity for their G34C variant compared to the starting Ta aptamer. While the EMSA experiments do have significant flaws, the observations of relative relationships in equilibrium binding affinities among the tested aptamer variants can be interpreted with reasonable confidence, given that they were all performed in a consistent manner.

We sincerely appreciate your valuable concerns and constructive feedback, which have greatly facilitated the improvement of our manuscript. Regarding the flaws of the EMSA experiments you pointed out, we have provided a detailed response to clarify the related issues and supplemented necessary experimental details to enhance the rigor and reproducibility of our work (see corresponding answers in the point-to-point response letter). It is worth noting that EMSA remains a classic and widely used technique for studying biomolecular interactions, and its reliability in qualitative and semi-quantitative analysis of binding events has been well recognized in the field. Furthermore, we fully agree with and are grateful for your view that, since all tested aptamer variants were analyzed using a consistent experimental protocol, the observations on the relative relationships of their equilibrium binding affinities can be interpreted with reasonable confidence. This recognition reinforces the validity of the relative affinity improvements we observed for the G34C variant compared to the parental Ta aptamer, which is a key finding of our study.

(4) The claim that the structure of the RBD-Aptamer complex predicted by the CAAMO pipeline is reliable is tenuous. The success of their rational design approach based on the structure predicted by several ensemble approaches supports the interpretation of the predicted structure as reasonable, however, no experimental validation is undertaken to assess the accuracy of the structure. This is not a main focus of the manuscript, given the applied nature of the study to identify Ta variants with improved binding affinity, however the structural accuracy claim is not strongly supported without experimental validation (i.e. chemical footprinting methods).

We thank the reviewer for this comment and agree that experimental validation would be required to establish the structural accuracy of the predicted RBD–aptamer complex. We note, however, that the primary aim of this study is not structural determination, but the development of a general computational framework for aptamer affinity maturation. In most practical applications, experimentally resolved structures of aptamer–protein complexes are unavailable. Accordingly, CAAMO is designed to operate under such conditions, using computationally generated binding models as working hypotheses to guide rational optimization rather than as definitive structural descriptions. In this context, the predicted structure is evaluated by its utility for affinity improvement, rather than by direct structural validation. We have revised the manuscript to clarify this scope.

The following highlighted words have been added to the revised manuscript.

“We note that CAAMO is not intended to establish experimentally validated complex structures, but rather to provide preliminary binding models that enable rational affinity maturation of aptamers in scenarios where structural information is limited or unavailable.”

“Overall, these results indicate that the proposed binding conformation of the aptamer Ta to the RBD serves as a plausible working binding model for structure-guided aptamer optimization, and demonstrate the great potential of our CAAMO framework in aptamer design and optimization.”

“which supports the robustness of our approach in generating informative binding models for comparative analysis and affinity optimization of an RNA aptamer with a target protein.”

“We believe that the predicted binding conformation represents a plausible member of the predicted ensemble that is functionally informative for guiding structure-based aptamer optimization, although it may not correspond to the exact native structure.”

(5) Throughout the manuscript, the phrasing of "all tested antibodies" was used, despite there being only one tested antibody in experimental methods and three distinct antibodies in computational methods. While this concern is focused on specific language, the major conclusion that their designed aptamers are as good or better than neutralizing antibodies in general is weakened by only testing only three antibodies through computational binding measurements and a fourth single antibody for experimental testing. The contact residue mapping furthermore lacks clarity in the number of structures that were used, with a vague description of structures from the PDB including no accession numbers provided nor how many distinct antibodies were included for contact residue mapping.

We thank the reviewer for this important comment regarding language precision, experimental scope, and clarity of the antibody dataset used in this study. We agree that the phrase “all tested antibodies” was imprecise and could lead to overgeneralization. We have carefully revised the manuscript to use more accurate and explicit wording throughout, clearly distinguishing between experimentally tested antibodies, computationally analyzed antibodies, and antibody structures used for large-scale contact analysis.

Specifically, the experimental comparison in this study was performed using one commercially available SARS-CoV-2 neutralizing antibody, whereas free energy–based computational analyses were conducted on three representative neutralizing antibodies with available structural data. We have revised the text to explicitly state these distinctions and have avoided general statements referring to neutralizing antibodies as a class.

Importantly, the residue-level contact frequency analysis was not based solely on these individual antibodies. Instead, this analysis leveraged a comprehensive set of experimentally resolved SARS-CoV-2 RBD–antibody complex structures curated from the Coronavirus Antibody Database (CoV-AbDab), a publicly available and actively maintained resource developed by the Oxford Protein Informatics Group. CoV-AbDab aggregates all published coronavirus-binding antibodies with associated PDB structures and provides a systematic and unbiased structural foundation for antibody–RBD interaction analysis. All available high-resolution RBD–antibody complex structures indexed in CoV-AbDab at the time of analysis were included to compute contact residue frequencies across the structural ensemble. We have now explicitly stated this data source, clarified the number and nature of structures used, and added the appropriate citation (Raybould et al., Bioinformatics, 2021, doi: 10.1093/bioinformatics/btaa739).

Finally, we have revised the conclusions to avoid claims that extend beyond the scope of the data. The comparison between aptamers and antibodies is now framed in terms of representative antibodies and consensus interaction patterns derived from a large structural ensemble, rather than as a general statement about all neutralizing antibodies. These revisions improve the clarity, rigor, and reproducibility of the manuscript, while preserving the core conclusion that the CAAMO framework enables effective structure-guided affinity maturation of RNA aptamers.

The following highlighted words have been added to the revised manuscript.

“Notably, the aptamer Ta^G34C^ exhibited the highest binding affinity to the RBD, outperforming the tested neutralizing antibodies in competitive binding assays.”

“Since we determined the most probable binding model of the aptamer Ta to the RBD, comparing the binding properties of the aptamer Ta with those of representative neutralizing antibodies to the RBD is both feasible and meaningful.”

“To further explore this, we analyzed the contact ratios of residues on the RBD bound to ACE2 (derived from MD simulations), to the aptamer Ta (derived from MD simulations), or to the neutralizing antibodies (derived from all available experimentally resolved SARS-CoV-2 RBD–antibody complex structures curated in the Coronavirus Antibody Database, CoV-AbDab [35]). CoV-AbDab is a publicly available, curated database that aggregates all published coronavirus-binding antibodies with associated structural information, providing a comprehensive and unbiased structural ensemble for contact frequency analysis.”

“Notably, the Ta-RBD complex formation remained unchanged after adding the antibody (Fig. 3E), suggesting that the aptamer Ta exhibits binding capability comparable to the tested monoclonal neutralizing antibody.”

“neutralizing antibodies (derived from all available SARS-CoV-2 RBD–antibody complex structures curated in CoV-AbDab).”

“Our computational and experimental studies showed that the aptamer Ta has comparable binding abilities to the RBD compared to representative neutralizing antibodies analyzed in this study.”

Overall, the manuscript by Yang et al presents a valuable tool for rational design of improved RNA aptamer binding affinity toward target proteins, which the authors call CAAMO. Notably, the method is not intended for de novo design, but rather as a tool for improving aptamers that have been selected for binding affinity by other methods such as SELEX. While there are significant issues in the conclusions made from experiments in this manuscript, the relative relationships of observed affinities within this study provide solid evidence that the CAAMO framework provides a valuable tool for researchers seeking to use rational design approaches for RNA aptamer affinity maturation.

**Recommendations for the authors:**

**Reviewer #4 (Recommendations for the authors):**

The computational aspects seem to be the strength of this manuscript, however there remain some issues with experimental approaches. The previous reviewers concern with non-specific binding remains an issue that should be dealt with through additional experimentation. The indication of Tc showing no binding is a good control for nonspecific RNA binding by RBD, but does not address nonspecific protein binding by Ta or its derivatives. For example, if a variant of Ta bound strongly to hydrophobic or highly charged patches in binding sites, they could also bind strongly to hydrophobic or highly charged patches in other proteins. As such, a non-specific binding test should be included for all tested variants to show target-specific binding.

Thank you for your constructive suggestion. To address the concern of non-specific binding, we have supplemented a dedicated control experiment using bovine serum albumin (BSA) as the non-specific protein target. The results demonstrated that Ta and its derivatives exhibited specific binding to the RBD protein. Detailed experimental procedures and corresponding results for this control assay are provided in our response to your first comment in this point-by-point response letter.

There is a serious concern to me that all data (i.e. the triplicate EMSAs claimed in your study) are not shown, with only one EMSA replicate shown for each variant in the supplemental materials. Additionally, the manuscript does not include unedited gel images, with apparent splicing of images in Figure 4E. All raw data should be available for review, which includes unedited images of the entirety of each gel electrophoresis experiment. Moreover, internal controls (positive of Ta+/-RBD, negative of Tc+/-RBD, and aptamer+/-non-RBD-protein) should be included and shown in every EMSA experiment.

Thank you for raising these critical concerns regarding the rigor and completeness of our EMSA experimental data. We highly appreciate your attention to detail, which helps us improve the quality and transparency of our manuscript.

First, regarding the number of EMSA replicates, we have indeed performed triplicate EMSA experiments for each variant, and all three replicates are provided in the supplementary materials (Fig. S9 of the original manuscript). We have added explicit labels for each replicate in the revised Fig. S9 to avoid confusion, ensuring the reproducibility of our results is clearly demonstrated.

Second, concerning unedited gel images, we fully agree with the importance of providing uncropped, raw gel images for peer review. In the revised manuscript, all unedited, full-length raw images of each gel electrophoresis experiment have been included in Supplementary Fig. S12, with clear annotations to correspond to the cropped images in the main text.

Third, with respect to internal controls, we acknowledge the necessity of comprehensive internal controls for EMSA experiments to validate specific binding. For the EMSA assays of RBD with Ta and its variants (Fig. 4E), we have already included the full set of internal controls, namely the Ta-RBD positive control, Tc-RBD negative control, and non-RBD protein control. Notably, the K_d_ values of RBD binding to Ta, Tc, and Ta variants are consistent with the signal intensity exhibited in the EMSA images, which further corroborates the reliability of our binding results. In addition, we have supplemented non-specific binding control data in the revised Supplementary Fig. S10, which fully validates the binding specificity between Ta/its derivatives and RBD and effectively rules out non-specific binding.
